# dsPIG: a tool to predict imprinted genes from the deep sequencing of whole transcriptomes

**DOI:** 10.1186/1471-2105-13-271

**Published:** 2012-10-19

**Authors:** Hua Li, Xiao Su, Juan Gallegos, Yue Lu, Yuan Ji, Jeffrey J Molldrem, Shoudan Liang

**Affiliations:** 1 Shanghai Center for Systems Biomedicine, Key Laboratory of Systems Biomedicine (Ministry of Education), Shanghai Jiao Tong University, Shanghai, 200240, China; 2 Department of Stem Cell Transplantation and Cellular Therapy, The University of Texas M D Anderson Cancer Center, Houston, TX, 77030, USA; 3 Division of Biostatistics, The University of Texas School of Public Health at Houston, Houston, TX, 77030, USA; 4 Department of Molecular and Human Genetics, Baylor College of Medicine, Houston, TX, 77030, USA; 5 Department of Leukemia, The University of Texas MD Anderson Cancer Center, Houston, TX, 77030, USA; 6 Center for Clinical and Research Informatics, NorthShore University HealthSystem, Chicago, Il, 60201, USA; 7 Department of Bioinformatics and Computational Biology, The University of Texas MD Anderson Cancer Center, Houston, TX, 77030, USA

**Keywords:** Prediction of imprinted genes, Transcriptome deep sequencing, mRNA-Seq, Bayesian model, Analysis of allelic expression

## Abstract

**Background:**

Dysregulation of imprinted genes, which are expressed in a parent-of-origin-specific manner, plays an important role in various human diseases, such as cancer and behavioral disorder. To date, however, fewer than 100 imprinted genes have been identified in the human genome. The recent availability of high-throughput technology makes it possible to have large-scale prediction of imprinted genes. Here we propose a Bayesian model (dsPIG) to predict imprinted genes on the basis of allelic expression observed in mRNA-Seq data of independent human tissues.

**Results:**

Our model (dsPIG) was capable of identifying imprinted genes with high sensitivity and specificity and a low false discovery rate when the number of sequenced tissue samples was fairly large, according to simulations. By applying dsPIG to the mRNA-Seq data, we predicted 94 imprinted genes in 20 cerebellum samples and 57 imprinted genes in 9 diverse tissue samples with expected low false discovery rates. We also assessed dsPIG using previously validated imprinted and non-imprinted genes. With simulations, we further analyzed how imbalanced allelic expression of non-imprinted genes or different minor allele frequencies affected the predictions of dsPIG. Interestingly, we found that, among biallelically expressed genes, at least 18 genes expressed significantly more transcripts from one allele than the other among different individuals and tissues.

**Conclusion:**

With the prevalence of the mRNA-Seq technology, dsPIG has become a useful tool for analysis of allelic expression and large-scale prediction of imprinted genes. For ease of use, we have set up a web service and also provided an R package for dsPIG at http://www.shoudanliang.com/dsPIG/.

## Background

Diploid eukaryotic species inherit two copies (i.e., two alleles) of the same gene from both parents. If one allele fails to work properly, the other allele can still implement a gene’s cellular function. For some genes, however, this protective mechanism is disabled because only one allele is expressed and its failure probably leads to cellular malfunction. These monoallelically expressed genes can be classified into one of three categories [1]: X-inactivated genes, which are regulated by a random process in which one of the two X chromosomes present in female mammals is silenced [2]; autosomal genes subject to random monoallelic gene expression, such as the T cell receptors and natural killer cell receptors [3-9]; and autosomal imprinted genes (e.g., *CDKN1C* and *H19*), which express from only one of the two alleles according to their parental origin [10-13]. Imprinted genes play important functional roles in the control of embryonic growth and development, as well as in post-natal development [14-16]. As imprinted genes are expressed from only one of the two parental chromosomes, a de facto haploid state is caused by imprinting and leads to asymmetric functions of parental genomes and loss of diploid protection against recessive mutations [11]. Thus, imprinting dysregulation is linked to numerous human genetic diseases, such as developmental disorders (Prader-Willi syndrome, Angelman syndrome) and cancers (neuroblastoma, Wilms’ tumor) [17-20]. In addition, environmental factors can influence gene expression by targeting imprinted genes [21,22]. Because imprinted genes are more susceptible to disease than non-imprinted genes [23,24], it is of great importance to identify novel imprinted genes for human.

Identifying imprinted genes experimentally has traditionally been a slow process, and the number of validated ones is much lower than the previous estimation (~1% of all human genes) [25]. However, recently developed high-throughput screening approaches (e.g., expression profiling and single-nucleotide polymorphism [SNP] microarrays) and recently identified DNA sequence characteristics (e.g., the number and type of repeated elements flanking a gene) have led to the proposal of several new methods to predict imprinted genes on a global scale [26-31]. With advances in next-generation sequencing technology [32,33], mRNA-Seq is becoming a powerful tool for transcriptome profiling [34]. It can generate not only the number of reads mapped to exons, which reflects the expression levels of a gene, but also the actual sequence, which may identify the allele from which the mRNA is expressed. Therefore, inference can be made for predicting imprinted genes. For example, by sequencing whole transcriptomes from mice embryos, Babak *et al.* (2008) measured allelic expression bias and identified six novel imprinted genes [35].

However, to our knowledge, prediction of imprinted genes by deeply sequencing transcriptomes (mRNA-Seq) from multiple independent tissues is still an open problem. In this study, we proposed a Bayesian model – dsPIG (deep sequencing-based Prediction of Imprinted Genes) – to predict imprinted genes based on allelic expression inferred from observed SNPs in mRNA-Seq data of independent human tissues. With dsPIG, we were able to measure the imbalance of allelic expression among various tissues and calculate the posterior probability of imprinting status for each gene. Under a stringent probabilistic cut-off of the posteriors and other reasonable biological criteria, we identified 57 potentially imprinted genes from 9 diverse human tissues and 94 potentially imprinted genes from 20 cerebellar cortices, with an expected low false discovery rate (FDR). Furthermore, analysis of allelic expression of the same genes among different tissues revealed that, in some cases, even if a gene was biallelically expressed, one allele always had higher expression level than the other.

## Results

### Statistical model development

Monoallelic expression generally falls into one of three categories: imprinted expression, random monoallelic expression and X-inactivation, all of which express only one of two alleles in a single cell [1-10]. At a tissue level, however, random monoallelic expression will allow both alleles to be detected in total RNA because of the “mosaicism” of the tissue [9,36] (also see discussion). Because our study was based on whole transcriptomes of tissue samples, random monoallelic expression was reasonably considered as biallelic expression when averaged over the entire tissue. X-inactivation was also excluded from this study by discarding all predictions on the X chromosome. Thus imprinting is the most likely cause of the observed monoallelic expression among transcriptomes of different tissues even though we cannot infer the parent of origin.

We used known SNPs from dbSNP [37] to distinguish and count the two alleles of each gene. If a gene was imprinted, we expected to observe only one of the two alleles of each SNP in the exon region from the whole transcriptome. With the allelic counts obtained from the mRNA-Seq data (see Materials and Methods), we developed a Bayesian model (dsPIG) to compute the posterior probability of imprinting based on each single SNP. Suppose we have sequenced transcriptomes from *n* independent tissue samples. For each sample, we count the alleles of all known SNPs, discarding those with 0 counts. For each SNP, let the allelic counts be: (*x*_*1*_*, y*_*1*_), (*x*_*2*_*, y*_*2*_)…, (*x*_*n*_*, y*_*n*_), where *x*_*k*_ and *y*_*k*_ are the counts for two alleles *X* and *Y* in the sample *k* (*k=*1*, …, n*). Because each gene can only have two statuses: imprinted (*I*) or non-imprinted (*NI*), we consider (*I*, *NI*) as a binary event vector for the imprinting status. By denoting *data =* {(*x*_*1*_*, y*_*1*_), (*x*_*2*_*, y*_*2*_)…, (*x*_*n*_*, y*_*n*_)}, we have by Bayes’ Theorem:

(1) $$\mathrm{Pr}\left(I|data\right)=\frac{\left(\mathrm{Pr}data|I\right)\times \mathrm{Pr}\left(I\right)}{\mathrm{Pr}\left(data|I\right)\times \mathrm{Pr}\left(I\right)+\mathrm{Pr}\left(data|NI\right)\times \mathrm{Pr}\left(NI\right)}$$

where Pr(*I* | *data*) is the posterior probability of imprinting and Pr(*I*) is the prior of imprinting. Based on current knowledge of prevalence of imprinted genes [25], we set the prior Pr(*I*)=1%, thus Pr(*NI*) = 1 – Pr(*I*) = 99%. Since samples were independent of each other and genotype has only 3 possible combinations (*XX, XY, YY*), we denote the genotype as follows:

$$\delta =\{\begin{array}{l} 1\;\text{the genotype is}\;XX\\ 2\;\text{the genotype is}\;XY\\ 3\;\text{the genotype is}\;YY \end{array}$$

Assuming *p* and *q* are the allele frequencies for allele *X* and *Y*, *p* + *q* =1. According to Hardy-Weinberg equilibrium, the prior probabilities for the three genotypes are calculated as follows:

$$\mathrm{Pr}\left(\delta \right)=\{\begin{array}{l} =\mathrm{Pr}{\left(X\right)}^{2}=p^{2}\;\delta =1\\ =\mathrm{Pr}(X)\mathrm{Pr}(Y)=2pq\;\delta =2\\ =\mathrm{Pr}{\left(Y\right)}^{2}=q^{2}\;\delta =3 \end{array}$$

Since values of *p* and *q* can be retrieved from dbSNP [37], *p* and *q* are treated as constants.

We used the law of total probability to calculate the likelihood Pr(*data*|*I*) as follows:

(2) $$\mathrm{Pr}\left(\text{data|}I\right)=\prod_{k=1}^{n}\mathrm{Pr}\left(x_{k},y_{k}|I\right)\;\left(n\geq 1\right)$$

$$\begin{array}{l} =\prod_{k=1}^{n}\left[\mathrm{Pr},\left(x_{k},y_{k}|I,\delta =1\right),\times ,p^{2},+,\mathrm{Pr},\left(x_{k},y_{k}|I,\delta =2\right)\right)\\ \;\left(\times ,2,p,q,+,\mathrm{Pr},\left(x_{k},y_{k}|I,\delta =3\right),\times ,q^{2}\right] \end{array}$$

By assuming that (*i*) the transcript levels of a gene’s two alleles are equal if the gene is biallelically expressed, (*ii*) two different alleles, if both expressed, have the same chance to be sequenced by mRNA-Seq, and (*iii*) unexpressed alleles with counts >0 are due to sequencing errors, we had the following derivation:

(3) $$\mathrm{Pr}\left(x_{k},y_{k}|I,\delta =1\right)=f\left(y_{k};n_{k},p_{e}\right)$$

(4) $$\mathrm{Pr}\left(x_{k},y_{k}|I,\delta =3\right)=f\left(x_{k};n_{k},p_{e}\right)$$

$$\mathrm{Pr}\left(x_{k},y_{k}|I,\delta =2\right)$$

$$\begin{array}{l} =\mathrm{Pr}\left(x_{k},y_{k}|I,\delta =2,\theta =0\right)\times \mathrm{Pr}\left(\theta =0\right)\\ \;+\text{Pr}\left(x_{k},y_{k}|I,\delta =2,\theta =1\right)\times \text{Pr}\left(\theta =1\right) \end{array}$$

(5) $$=f\left(y_{k};n_{k},p_{e}\right)\times \frac{1}{2}+f\left(x_{k};n_{k},p_{e}\right)\times \frac{1}{2}$$

Here, *f* denotes binomial distribution [i.e., $f\left(x;n,p\right)=\left(\begin{array}{l} n\\ x \end{array}\right){\left(1-p\right)}^{\left(n-x\right)}p^{x}$], *n*_*k*_ = *x*_*k*_ + *y*_*k*_ is assumed fixed, and *p*_*e*_ is the averaged sequencing error rate for each SNP (*p*_*e*_ was obtained from Wang *et al.* 2008). The binary variable θ is defined as follows $\theta =\{\begin{array}{l} 0\;\text{Only}\;X\;\text{can be expressed due to imprinting}\\ 1\;\text{Only}\;Y\;\text{can be expressed due to imprinting} \end{array}$. Since *X* and *Y* have an equal chance to be inherited from either maternal or paternal genome, *X* and *Y* have an equal chance to be expressed in imprinted genes. Hence, $\mathrm{Pr}\left(\theta \right)=\{\begin{array}{l} 1/2\;\theta =0\\ 1/2\;\theta =1 \end{array}$.

Similarly for the likelihood function Pr(*data*|*NI*), we have:

(6) $$\mathrm{Pr}\left(data|NI\right)=\prod_{k=1}^{n}\mathrm{Pr}\left(x_{k},y_{k}|NI\right)\;\left(n\geq 1\right)$$

$$\begin{array}{l} =\prod_{k=1}^{n}\left[\mathrm{Pr},\left(x_{k},y_{k}|NI,\delta =1\right),\times ,p^{2},+,\mathrm{Pr},\left(x_{k},y_{k}|NI,\delta =2\right)\right)\\ \;\left(\times ,2,p,q,+,\mathrm{Pr},\left(x_{k},y_{k}|NI,\delta =3\right),\times ,q^{2}\right] \end{array}$$

(7) $$\mathrm{Pr}\left(x_{k},y_{k}|NI,\delta =1\right)=f\left(y_{k};n_{k},p_{e}\right)$$

(8) $$\mathrm{Pr}\left(x_{k},y_{k}|NI,\delta =3\right)=f\left(x_{k};n_{k},p_{e}\right)$$

Based on our three assumptions (*i*) – (*iii*), Pr(*x*_*k*_, *y*_*k*_|*NI*, *δ* = 2) follows a binomial distribution with *p*=0.5. Therefore, we have:

(9) $$\mathrm{Pr}\left(x_{k},y_{k}|NI,\delta =2\right)=\left(\begin{array}{l} x_{k}+y_{k}\\ \;x_{k} \end{array}\right){\left(\frac{1}{2}\right)}^{x_{k}+y_{k}}$$

Computation is performed separately for each single SNP. Therefore, a posterior probability of imprinting for a gene is associated with a specific SNP in this gene, unless otherwise specified.

### Simulation-based model analysis

**Figure 1:**
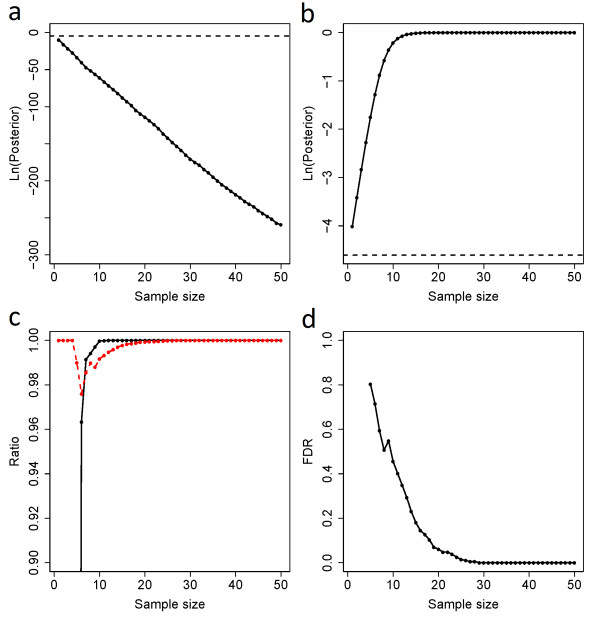
**Simulation-based performance analysis of dsPIG.a**, **b**, Simulated (natural log-transformed) posteriors of (**a**) biallelically expressed genes and (**b**) imprinted genes. The dashed line in both panels stands for the log-transformed prior (0.01). Results in (**a**) and (**b**) were based on 20,000-time simulations with geometric mean as the method of averaging posteriors. **c**, Sensitivity (the black solid line) and specificity (the read dashed line) of our model. **d**, the FDR of our predictions. When sample size was <5, the FDR was not computable as sensitivity and specificity were both 0. Results in (**c**) and (**d**) were based on 20,000-time simulations with geometric mean as the method of averaging posteriors.

**Figure 2:**
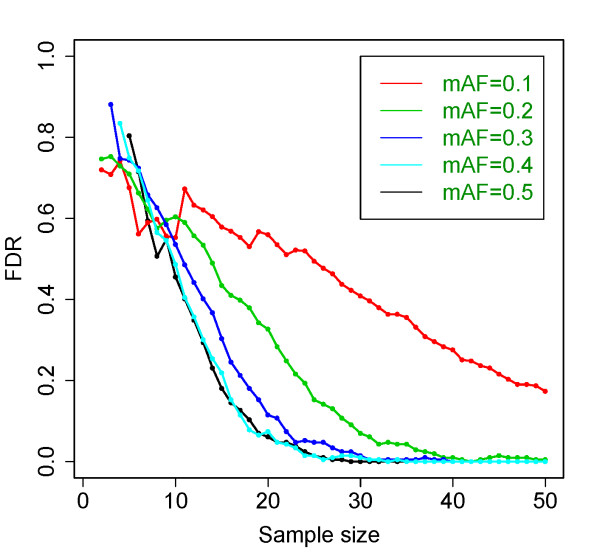
**FDRs of our predictions with respect to different allele frequencies.** When minor allele frequency (mAF) decreased from 0.5 to 0.1, FDR generally increased if sample size was >10. Results were based on 20,000-time simulations. For detailed values of FDR, please refer to Additional file 4: Table S2.

We generated allelic counts from simulated data by taking into account imprinting status, SNP frequency, and sequencing error. We then applied dsPIG to estimate the sensitivity, specificity and the FDR. We generated two sets of allelic counts under the assumption that the locus was either imprinted or not. The number of reads for each allele was generated assuming the presence of one dominating allele plus sequencing error for imprinted case; and presence of equal amount of two alleles plus sequencing error for non-imprinted case. The generated allelic counts followed a distribution similar to the actual mRNA-Seq data (Additional file 1 Figure S1). More details of the procedure were illustrated in Additional file 2 Figure S2. Given an allele frequency (0.5), a sequencing error (0.02) and a prior (0.01) of imprinting, the posterior probability calculated from the allelic counts generated for imprinted genes approached 1 as the sample size increased, while concomitantly the posterior probability for biallelically expressed approached 0 (Figure 1a,b; for each SNP, only the tissue samples with allelic counts >2 were considered valid samples, and we used “sample size” to refer to the number of valid samples in this study). With minor allele frequencies between 0.005 to 0.5, sequencing errors between 0.01 to 0.05 and priors between 0.005 to 0.02, we obtained similar results as Figure 1a,b. Using 0.2 as the cut-off for posteriors Pr(*I* | *data*) (see Model Analysis Based on Independent Test Sets), sensitivity of our model-based prediction exceeded 99.9% when sample size was >9, and specificity exceeded 99.99% when sample size was >18 (Figure 1c). Under the same cut-off and the allele frequency, the FDR of predicted imprinted genes decreased as sample size increased: it dropped to 5% and 1% as sample size exceeded 20 and 25, respectively (Figure 1d). Using the same cut-off (0.2), we also examined how FDR varied when minor allele frequency changed from 0.1 to 0.5 and sample size increased from 1 to 50 (Figure 2). Based on these simulations, we were able to provide estimated FDRs for most of our predictions in this study (Additional file 3 Table S1).

### Predictions of imprinted genes

**Figure 3:**
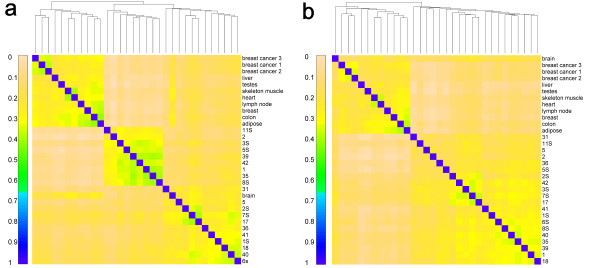
**Sample clustering in terms of imprinting-inclined SNPs.** Spearman correlations were calculated between each pair of samples using the posterior on each SNP calculated by dsPIG in each sample. Hierarchical clustering was conducted with average linkage as the agglomerative method. Posterior probabilities of African American samples were computed with African American allele frequency in panel (**a**) and with Caucasian allele frequency in panel (**b**).

We collected two previously published mRNA-Seq data sets. One set included 9 diverse tissue samples (Group I) from Wang *et al.* 2008, and the other set had 20 cerebellum cortex samples (Group II) from Mudge *et al.* 2008 (Table 1; see Data Collection in Materials and Methods) [38,39]. Wang *et al.* 2008 showed that, in terms of alternative isoform expression, cerebellum tissues were clustered together and the 9 diverse samples were more closely correlated. Here we performed hierarchical clustering based on the imprinting-inclined SNPs (i.e., SNPs with posteriors >0.01) and obtained similar results (Figure 3; see Sample Clustering in Materials and Methods). As we expected, samples from the cerebellar cortex were clustered together, with Caucasian and African American separated in two sub-clusters (Figure 3a). Using Caucasian allele frequency on African American samples, however, yielded a sub-cluster without separation between the two ethnicities (Figure 3b). This suggests that the separation observed in Figure 3a was due to differences in minor allele frequencies. As a test set, 3 breast cancer cell line samples were clustered together in both cases. Compared with other non-cerebellum samples, the brain sample had higher correlation with cerebellum samples in both cases, which is sensible biologically. The result that the 9 diverse tissue samples were clustered together could be caused by many factors such as different experimental conditions between Group I and Group II samples.

**Table 1:** Sample information and sequencing results from 9 various normal tissues (Group I) and 14 cerebellar cortices with schizophrenia (SCZD) and 6 normal cerebellar cortices (Group II)

	**Sample**	**Type**	**Ethnicity**	**Average read length**	**Number of reads**	**Unique genomic reads**
Group I	Adipose	Normal	Caucasian	32	27752231	63%
	Brain	Normal	Caucasian	32	17246957	64%
	Breast	Normal	Caucasian	32	16120746	66%
	Colon	Normal	Caucasian	32	28435996	62%
	Heart	Normal	Caucasian	32	20169301	56%
	Liver	Normal	Caucasian	32	18517121	62%
	Lymph node	Normal	Caucasian	32	27492254	57%
	Skeletal muscle	Normal	Caucasian	32	22640454	64%
	Testes	Normal	Caucasian	32	27303938	68%
Group II	Cerebellum 1	SCZD	African American	32	23241938	68%
	Cerebellum 11S	SCZD	African American	35	14572861	54%
	Cerebellum 18	SCZD	Caucasian	32	25129004	69%
	Cerebellum 1S	SCZD	Caucasian	32	36760977	64%
	Cerebellum 2	SCZD	African American	36	19241726	60%
	Cerebellum 31	SCZD	Caucasian	36	19867823	63%
	Cerebellum 36	SCZD	Caucasian	32	20111871	67%
	Cerebellum 39	SCZD	African American	33	23055778	63%
	Cerebellum 3S	SCZD	African American	34	17846750	46%
	Cerebellum 41	SCZD	Caucasian	32	38658913	66%
	Cerebellum 42	SCZD	African American	35	17588723	56%
	Cerebellum 5	SCZD	Caucasian	36	21229299	61%
	Cerebellum 5S	SCZD	African American	32	28944566	66%
	Cerebellum 7S	SCZD	Caucasian	34	13769073	54%
	Cerebellum 17	Normal	Caucasian	36	12890252	47%
	Cerebellum 2S	Normal	Caucasian	36	12482759	44%
	Cerebellum 35	Normal	African American	36	25402905	63%
	Cerebellum 40	Normal	Caucasian	36	24486091	64%
	Cerebellum 6S	Normal	Caucasian	32	24347196	71%
	Cerebellum 8S	Normal	African American	32	24016465	71%

Using dsPIG, we predicted imprinted genes for Group I and Group II separately. To call a gene imprinted, we checked the posteriors of all the SNPs on the same gene to make sure that (*i*) the gene had at least one SNP with a posterior >0.2, which was the same cut-off used in simulations, and (*ii*) all the other SNPs did not show any contradictory evidence (i.e., all the other posteriors were >0.002 [our 20,000-time simulations showed that 95% of the posteriors of imprinted genes were >0.002]). After applying dsPIG to the mRNA-Seq data and using the above criteria, we predicted 57 potentially imprinted genes for Group I samples and 94 potentially imprinted genes for Group II samples out of a total of 20,559 genes (0.28% and 0.46%, respectively) that had allele frequency data in dbSNP. The distribution of sample sizes for SNPs was shown in Additional file 5 Figure S3. We listed the top 15 predictions for both groups with respect to their posterior probabilities of imprinting in Table 2 (see Additional file 3 Table S1 for the complete list). Surprisingly, we found only 13 common genes between the 57-gene list and the 94-gene list. Functional enrichment analysis performed in Ingenuity® Pathway Analysis (IPA) showed that the two lists of genes were significantly enriched (p-value<0.05 after “BH” correction [40]) in certain Bio Functions but not in any canonical pathways defined by IPA (Additional file 6 Figure S4). We also compared our predictions with the 371 genes that are subject to random monoallelic expression [1] and found none of our predicted genes in either group overlapped with them, which further validates the quality of our predictions and strongly suggests that our predictions are not affected by random monoallelic expression.

**Table 2:** The top 30 predictions of imprinted genes based on mRNA-Seq data from Group I and Group II samples

**SNP(rs#)**	**Chr**	**Location**	**Str**	**Posterior**	**GeneID**	**Symbol**	**SS**	**FDR**	**Group**
11538691	chr17	4789783	+	1	5216	PFN1	9	0	I
178412	chr7	73173272	-	1	3984	LIMK1	8	0	I
17094371	chr14	57677831	+	1	145407	C14orf37	9	0	I
2596331	chr1	143820905	-	0.999996	9554	SEC22B	7	0.26	I
8110904	chr19	47723208	+	0.999995	634	CEACAM1	4	0	I
11555395	chr17	67629054	+	0.999993	6662	SOX9	5	0	I
4015375	chr7	89628110	+	0.99997	26872	STEAP1	6	0	I
4015375	chr7	89628110	+	0.99997	256227	MGC87042	6	0	I
10208923	chr2	141157767	+	0.9998	53353	LRP1B	5	0	I
9807047	chr17	46229893	+	0.999751	731414	LOC731414	7	0	I
584959	chr3	61703763	+	0.995669	100128936	RPL10AP6	9	0.052	I
10800864	chr1	201003241	+	0.995398	10765	KDM5B	9	0.052	I
10306	chr10	74437407	-	0.993645	5033	P4HA1	4	0	I
2009646	chr5	108148856	+	0.989758	2241	FER	6	0	I
2009646	chr5	108148856	+	0.989758	643534	LOC643534	6	0	I
178412	chr7	73173272	-	1	3984	LIMK1	11	0.098	II
11538691	chr17	4789783	+	1	5216	PFN1	20	0.023	II
11541557	chr1	226352225	+	1	375	ARF1	9	0	II
17492855	chr2	158989711	+	0.999999	130940	CCDC148	18	0.014	II
2352731	chr3	144378737	+	0.999995	5089	PBX2	10	0.072	II
1065453	chr7	99755171	-	0.999992	441272	SPDYE3	5	0	II
11066116	chr12	110915447	+	0.999989	89894	TMEM116	8	0	II
2499	chr6	30021520	-	0.999982	3105	HLA-A	7	0.26	II
3093976	chr6	31610839	-	0.99993	7919	BAT1	8	0.12	II
705	chr15	22770604	+	0.999919	6638	SNRPN	20	0.023	II
705	chr15	22770604	+	0.999919	8926	SNURF	20	0.023	II
3132453	chr6	31712022	-	0.999913	7916	BAT2	4	0	II
1051470	chr12	117067614	+	0.999874	5037	PEBP1	14	0.051	II
1364261	chr16	70597171	-	0.999859	100130263	LOC100130263	8	0	II
11160608	chr14	100382845	+	0.999796	100130955	LOC100130955	20	0.023	II

Abbreviations: rs#-SNP identification number, Chr-chromosome, Str-strand, SS-sample size.

### Model analysis based on independent test sets

We collected 66 validated imprinted genes and 155 validated non-imprinted genes ([31,41]; see Data Collection) and used them to check for false-positive and false-negative predictions made by dsPIG. Based on the mRNA-Seq data of Group I samples, 28 of the 66 imprinted genes and 119 of the 155 non-imprinted genes had allelic counts for known SNPs; based on the mRNA-Seq data of Group II samples, 26 of the 66 imprinted genes and 110 of the 155 non-imprinted genes had allelic counts for known SNPs. Under the same criteria used to predict imprinted genes in Table 2, dsPIG identified 2 out of 28 imprinted genes from Group I samples and 9 out of 26 imprinted genes from Group II samples, based on the mRNA-Seq data; under the same criteria, dsPIG misidentified 0 out of 119 non-imprinted genes from Group I samples and 0 out of 110 non-imprinted genes from Group II samples. Moreover, among those imprinted genes, only 8 out of 28 (Group I) and 17 out of 26 (Group II) have sample sizes >5. On the contrary, all 11 dsPIG-identified imprinted genes (2 for Group I and 9 for Group II) have sample size >5. Therefore, for imprinted genes with sample size > 5, 2 out of 8 genes and 9 out of 17 genes could be identified by dsPIG for Group I and Group II, respectively. Again, this showed that the sensitivity of dsPIG increased as sample size increased and dsPIG could probably identify more imprinted genes if the number of sequenced tissue samples further increased. This also agreed with the simulation results. Interestingly, some of the validated imprinted genes had very small posteriors (<10^-8^), which indicated that they had biallelic expression (or random monoallelic expression) in certain tissues (Table 3).

**Table 3:** Tissues where validated imprinted genes most likely had biallelic expression

**SNP(rs#)**	**Chr**	**Location**	**Str**	**Posterior**	**Gene ID**	**Gene Symbol**	**Tissue**	**Group**
3807551	chr7	50627897	-	6.84E-17	2887	GRB10	Skeletal Muscle	I
2585	chr11	2107019	+	9.09E-210	3481	IGF2	Liver	I
10770125	chr11	2125589	+	7.15E-43	3481	IGF2	Liver	I
8813	chr11	3065081	-	1.20E-11	114879	OSBPL5	Colon	I
17178177	chr11	3065446	+	6.83E-10	114879	OSBPL5	Colon	I
10770125	chr11	2125589	+	7.15E-43	51214	IGF2AS	Liver	I
7121	chr20	56912201	+	0	2778	GNAS	Lymph node	I
854547	chr7	94761791	+	1.61E-10	55607	PPP1R9A	Cerebellum	II
8164	chr11	6372457	+	1.54E-30	6609	SMPD1	Cerebellum	II
7951904	chr11	6369506	+	2.68E-10	6609	SMPD1	Cerebellum	II
11601088	chr11	6371967	+	1.62E-09	6609	SMPD1	Cerebellum	II
2272666	chr8	1.46E+08	+	2.63E-17	79581	GPR172A	Cerebellum	II
2280840	chr8	1.46E+08	+	1.05E-57	79581	GPR172A	Cerebellum	II
2615374	chr8	1.41E+08	+	1.51E-13	51305	KCNK9	Cerebellum	II

Posteriors were calculated based on Group I or II (see Table 5). The “Tissue” column indicates where genes were most likely biallelically expressed. Genes with contradictory evidence (i.e., genes had posteriors <10^-8^ and posteriors ≥0.2) were discarded. IGF2, OSBPL5, SMPD1 and GPR172A had at least 2 SNPs with posteriors less than 10^-8^. Abbreviations are the same as in Table 2.

We used the same sets of imprinted and non-imprinted genes to determine the cut-off of the posteriors used in the prediction of imprinted genes. Because most genes (~99%) are expected to be non-imprinted, the cut-off has to yield a very high specificity (>99%) so that the overall FDR of our predictions can be low enough (<50%) for further validations. After trying different cut-offs (0.1, 0.2, …, 0.9), we found 0.2 to be the most appropriate cut-off in terms of the validated gene sets because (*i*) increasing the cut-off from 0.2 only lowered the sensitivity while left the specificity unchanged (~0%), and (ii) decreasing the cut-off from 0.2 lowered the specificity while the sensitivity didn’t change a lot, which substantially increased the FDR of predictions (Additional file 7: Figure S5). We also showed the ROC curves for dsPIG in Additional file 7: Figure S5.

### Candidates for experimental validation

We chose top 30 candidate genes from our predictions and listed them in Table 4. Except one SNP (rs#3106189), all the SNPs in Table 4 have high minor allele frequencies (>0.184), which indicates >30% chance of observing heterozygous alleles in experiments. In addition, these genes also met at least one of the following three criteria: (*i*) their SNPs (the 4^th^ column of Table 4) had a relatively low FDR (<0.3), (*ii*) they had multiple SNPs with posteriors > 0.2 (dsPIG calculated a posterior for each SNP in a gene), and (*iii*) they were located near existing imprinted genes (distance <2M base pairs) [10,42]. These additional criteria further increased the possibility of imprinting.

**Table 4:** Suggested predictions for experimental validation.

**Gene Symbol**	**Chr**	**Str**	**SNP(rs#)**	**Location**	**Posterior**	**SS**	**FDR**	**Group**
CCDC148	chr2	+	17492855	158989711	0.999999314	18	0.014	II
BAT1	chr6	-	3093976	31610839	0.999930191	8	0.12	II
PEBP1	chr12	+	1051470	117067614	0.999873859	14	0.051	II
**LOC100130955**	chr14	+	11160608	100382845	0.999795515	20	0.023	II
RPL10AP6	chr3	+	584959	61703763	0.999517203	11	0.055	II
KCNJ12	chr17	+	16962951	21259999	0.998953843	11	0.055	II
VARS2	chr6	+	1043483	31001706	0.998607936	14	0.051	II
BAT2	chr6	-	2272593	31709322	0.998223631	9	0.052	II
RPL10AP6	chr3	+	584959	61703763	0.995668725	9	0.052	I
KDM5B	chr1	+	10800864	201003241	0.995398014	9	0.052	I
TAPBP	chr6	-	1059288	33375649	0.995270134	13	0.057	II
KCNQ5	chr6	-	2000203	73753786	0.993101795	13	0.069	II
FER	chr5	+	2009646	108148856	0.989758091	6	0	I
LOC643534	chr5	+	2009646	108148856	0.989758091	6	0	I
LY6G5B	chr6	-	1266076	31748496	0.98719144	16	0.028	II
NOMO2	chr16	+	7179	14897344	0.979473344	7	0	II
BAT5	chr6	-	1475865	31765391	0.979015507	7	0	II
RXRB	chr6	-	6531	33271428	0.977747582	6	0	II
FER	chr5	+	2009646	108148856	0.962143895	6	0	II
ZBTB22	Chr6	-	1061783	33390605	0.96804794	12	0.076	II
LOC283398	chr12	-	3827521	93467186	0.908227908	7	0	I
UCRC	chr22	+	14115	28493525	0.889141351	15	0.039	II
MT2A	chr16	+	10636	55200843	0.709333922	10	0.142	II
RQCD1	chr2	+	526897	219141840	0.683651187	13	0.128	II
PSMC3IP	chr17	-	6963	37985122	0.661403579	12	0.146	II
**CSTF3**	chr11	-	1028564	33118584	0.479199531	8	0.373	II
**LOC100128252**	chr19	+	3971706	61697880	0.478128233	4	0.343	I
**LOC100130814**	chr14	-	2295655	100608883	0.446498085	18	0.228	II
**IMPDH1**	chr7	-	2228075	127821864	0.283348511	17	0.318	II
CLDN4	chr7	+	1127155	72884396	0.270700082	4	0.594	I

Boldface indicates proximity to known imprinted genes. Underline indicates a gene has multiple (≥3) SNPs with posteriors > 0.2. Genes are sorted by their posteriors. RPL10AP6 and FER are listed for both Group I and Group II. Abbreviations are the same as in Table 2.

### Detection of allele-preferred expression

By investigating the biallelically expressed genes identified in the mRNA-Seq data, we found that at least 18 genes expressed significantly more transcripts from one specific allele than the other among various individuals and tissues (*P* < 0.05 by binomial test; Table 5; see Binomial Test in Materials and Methods). This indicated that the difference between expression levels of the two alleles was not caused by sequencing errors or stochastic effects in RT-PCR. In future, as more mRNA-Seq data are generated, if more genes with one specific allele always under-expressed are observed, we would speculate that a sophisticated mechanism (such as nonsense-mediated mRNA decay [43]) may exist to explain this type of allelic preference in gene expression for biallelically expressed genes.

**Table 5:** 20 SNPs of which one specific allele had a higher transcript level than the other one among various tissues and individuals

**SNP**	**Chr**	**Position**	**Str**	**Ratio**	**P-value**	**Gene Symbol**
rs13884	chr19	18545100	+	1/24	3.58E-05	UBA52
rs4621	chr11	65380094	+	0/17	9.16E-05	CFL1
rs425485	chr19	3004801	+	0/13	0.000976563	AES
rs4874	chr17	17227958	-	0/12	0.001171875	LOC388344
rs6565924	chr18	72820212	+	0/12	0.001171875	MBP
rs6554	chr19	18546963	+	0/11	0.001953125	UBA52
rs11543289	chr17	34136120	+	0/10	0.003348214	MLLT6/LOC100129395
rs17626	chr19	44618360	-	1/13	0.005126953	RPS16
rs8118	chr16	4787169	-	0/9	0.005208333	ROGDI
rs9199	chr18	72821323	-	0/8	0.009375	MBP
rs7612	chr7	5533637	+	1/11	0.01171875	ACTB
rs6597982	chr11	778006	-	1/11	0.01171875	CEND1
rs1803283	chr14	1.03E+08	-	2/14	0.011944111	CKB
rs7982	chr8	27518397	-	2/13	0.015854779	CLU
rs3743566	chr16	57103285	+	2/13	0.015854779	NDRG4
rs2821	chr20	5853778	+	3/16	0.015854779	CHGB
rs7121	chr20	56912201	+	3/16	0.015854779	GNAS
rs12165042	chr17	30502328	+	4/18	0.020589193	UNC45B
rs1150	chr17	8003307	+	2/12	0.024362664	VAMP2
rs10064485	chr5	1.75E+08	+	2/11	0.039257813	CPLX2

The “Ratio” column shows the proportion of samples with one specific allele’s transcript level higher than the other’s. P-values have been adjusted by “BH” correction [40]. Abbreviations are the same as in Table 2.

### Web-based service and R package for dsPIG

We have provided a web-based service for dsPIG at http://www.shoudanliang.com/dsPIG/. Users need to upload either mapped mRNA-Seq data in the supported format or processed data files containing allelic counts for each SNP (see the website for more details). After uploading the data, users may set the values for (*i*) the cut-off for posteriors, (*ii*) the average sequencing error rate, (*iii*) the prior of imprinting, and (*iv*) QS (quality score). In addition, users need to specify the human genome build and the SNP build for dsPIG. Our server will calculate the posterior probabilities of imprinting for each gene and email the results back to the users. In the final submission form, users may request additional analysis, such as suggesting tissues where known imprinted genes most likely have biallelic expression. In addition, we have made an R package for dsPIG, which is available both on the website and in the supplementary materials (Additional file 8 for UNIX and Additional file 9 for Windows). The instruction and the sample files for this R package are in Additional file 10. The web service and the R package generate the same result on the predictions of imprinted genes. For reference, we have also provided the annotated code for dsPIG (including both the R code and the C code) used in this study as Additional file 11 and on the website.

## Discussion

**Figure 4:**
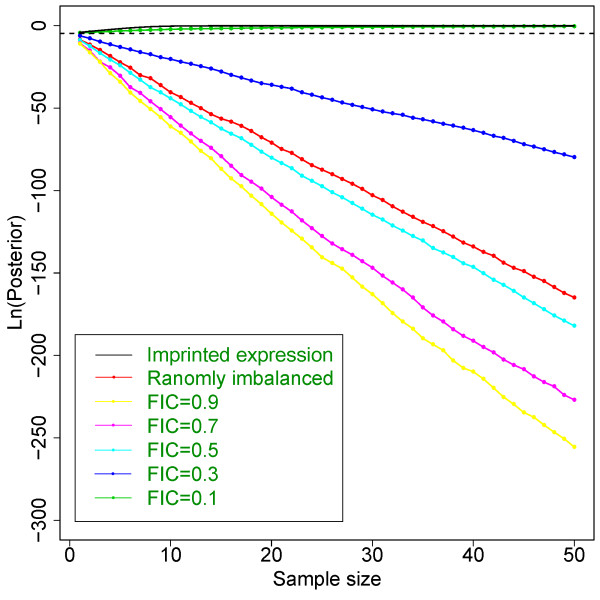
**Effect of imbalanced transcript levels on the posteriors of biallelically expressed genes.** Solid lines stand for simulated posteriors for imprinted genes (black line) and biallelically expressed genes (non-black lines). “Randomly imbalanced” means that in each sample we randomly picked one allele to have a lower expression level than the other allele. FIC indicates “Fixed Imbalanced Coefficient”, which means one allele is always expressed at a “FIC” level of the other one in all samples. The dashed line stands for the log-transformed prior. When FIC is low enough (typically <13%), posteriors are not able to tell the difference between imprinted (solid black line) and biallelic expression (green line).

dsPIG operates under the assumption that if a gene is biallelically expressed, the transcript levels of two alleles are the same. However, because imbalance of allelic expression has been widely detected in human tissues [27,44-46], our assumption may not always be correct. Indeed, within our 29-tissue samples, allelic imbalance was observed even after excluding possible expression from imprinted genes (data not shown here). However, our simulation studies showed that our model can still distinguish imprinted genes from non-imprinted ones in most situations (Figure 4), unless one specific allele is always expressed at <13% of the other allele’s expression level across different samples (no supporting literature for this yet). Stochastic RT-PCR amplification [4] is not of particular concern in dsPIG because this has been taken into account as allelic imbalances.

dsPIG is sensitive to biallelic expression and unlikely to falsely predict imprinted genes. A gene will get a very low posterior probability of imprinting when it obviously has biallelic expression in one tissue, even if only one allele of this gene is observed in the transcriptomes from all other tissues (Additional file 12: Figure S6). For example, a gene may have monoallelic expression because of allele-specific differences caused by either heterozygous SNPs or somatic mutations in the promoter region of the gene; as long as monoallelic expression caused by these conditions is not present population-wide and a large number (e.g., >25 in our study) of independent tissue samples is used in dsPIG, these genes will not be falsely predicted as imprinted genes. However, for the same reason, if transcriptomes are collected from various tissues, it becomes very hard for dsPIG to detect tissue-specific imprinted genes. This partially explains why dsPIG predicted much less imprinted genes in Group I samples than in Group II samples (another reason is that Group II has more samples than Group I).

One advantage of dsPIG is that it is able to predict imprinted genes without sequencing the genotype. Although a homozygous allele will also lead to identification of only one allele in the transcriptome, the result will not elevate the posterior belief of imprinting in our model. This is very important and practical for human because we no longer need to sequence the genome for genotypes. However, a disadvantage is that dsPIG cannot tell the parent of origin for predicted imprinted genes, which can be verified only by other studies.

One obvious limit of dsPIG is that it was modeled based on single SNPs. This means, if a gene has more than 1 SNP site in its exons, it may have different single-SNP-based allelic counts and thus different posterior probabilities from dsPIG. Therefore, one single strong posterior cannot determine the imprinting status of this gene. Instead, we have to look into all SNP sites of each gene and make sure no contradictory posterior exists for our predictions (as stated in results). In future, a possible improvement would be integration of all SNP information of a gene and calculate a single posterior to predict imprinting status.

## Conclusions

In this paper, we proposed a new method – dsPIG, applicable to all mammals with genomic imprinting, to predict imprinted genes based on mRNA-Seq data of various independent tissues. With enough sequenced samples, dsPIG is capable of predicting imprinted genes on a genome-wide scale with expected low FDRs. The power of dsPIG will be further enhanced after more data generated by mRNA-Seq technology become available in the near future.

## Methods

### Data collection

To predict gene imprinting status, we used ~650 million short reads from 29 human tissue samples [38,39]: 206 million from 9 different normal tissues, 320 million from 14 cerebellar cortices with schizophrenia and 124 million from 6 normal cerebellar cortices. Tissue samples were collected from 29 independent individuals, among which 20 were Caucasian and 9 were African American (Table 1). We downloaded SNP data from UCSC genome browser using the table function [47] and used SNPs with only two alleles to make predictions in this study. The source of the original data was SNP Database (dbSNP) build 129 from NCBI [37]. Known imprinted and non-imprinted genes were collected from Additional file 3: Table S1 and Additional file 4: Table S2 in the supplementary research data of Luedi *et al.* (2007) [31] and from Catalogue of Parent of Origin Effects (http://igc.otago.ac.nz/home.html) [41].

### Calculation of allelic counts

RNA-Seq reads were mapped to human genome hg18 from UCSC genome browser using Eland (GAPipeline-1.3.2). The unmapped reads were mapped to the exon-exon junctions downloaded from http://genes.mit.edu/burgelab/mrna-seq/[38]. The junctions contain 56 (28×2) base pairs in total, allowing the reads (32 base pairs) to be mapped with a minimum of 4 base pairs on each side of the junctions [38]. To compute the number of alleles for each SNP, we scanned each mapped tag for all known SNPs (in terms of dbSNP) and counted the number of times each nucleotide occurred at each SNP position in each sample. To reduce the amount of calculation, we only retained SNPs that were covered by any sequencing tag in any sample. This generated allelic counts for a total of 1,261,906 SNPs in the 29 tissue samples. We then discarded SNPs with unknown frequency and very low allelic counts (i.e., total allelic counts <3 in each sample). In addition, we defined a quality score-QS (see Definition of QS) and discarded SNPs with QS <0.9 in all 29 samples (see Additional file 13: Figure S7). After these steps, allelic counts for 82,916 SNPs remained, and these were used in dsPIG.

### Definition of QS

We only used biallelic SNPs in dbSNP, which is the majority of SNPs. Nucleotides observed other than these two (alleles) were considered as sequencing errors due to low sequencing quality at the SNP site, or indicted that the allelic information of the SNP was wrong in dbSNP. Thus, we defined quality score-QS to improve the quality of the SNPs used in our predictions:

$${\text{QS}}_{k}=\frac{x_{k}+y_{k}}{x_{k}+y_{k}+e_{k}}$$

In the above equation, *x*_*k*_ and *y*_*k*_ are the allelic counts of allele *X* and *Y* in the kth sample (*X* and *Y* were determined by dsSNP), and *e*_*k*_ is the count of additional nucleotide(s). QS was calculated for each SNP in each sample. We arbitrarily chose 0.9 as a cut-off for QS ( Additional file 13: Figure S7) and only those SNPs with QS>0.9 were used in dsPIG.

### Sample clustering

By applying dsPIG to each of the 29 samples, we obtained 29 lists of posterior probabilities (each list had 87,852 posteriors for 87,852 SNPs), which were first multiplied by 100 and then natural logarithm transformed. Thus, if the posterior was the same as the prior (0.01), it would become 0 after the transform. After that, for each SNP, all posteriors ≤ 0 were modified to 0, while all posteriors > 0 were kept at the same value. If all 29 posteriors for a SNP were 0, the SNP was removed from the 29 lists. By doing this, only the SNPs that showed an increased probability of imprinting were kept for clustering. We then computed spearman correlations between samples based on the remaining 29 lists of posteriors, used these correlation values to determine distance between samples, and performed hierarchical clustering in R (http://www.r-project.org). By using this method, we clustered samples in terms of imprinting-inclined SNPs, and thus reduced the influence of biallelically expressed genes.

### Binomial test

For a single SNP, we first defined $P_{k}=\frac{x_{k}}{y_{k}}$, where *x*_*k*_ and *y*_*k*_ are the allelic counts for its alleles *X* and *Y* in the sample *k* (*k=1, …, n*). We then defined $z=\sum_{k=1}^{n}I\left(P_{k}\right)$, where $I\left(P_{k}\right)=\{\begin{array}{l} 1,\;\text{if}\;P_{k}>1\\ 0,\;\text{if}\;P_{k}<1 \end{array}$. We used the following criteria to find the SNPs with both alleles (*XY*) expressed in the sample *k*: *x*_*k*_>10, *y*_*k*_>10, *x*_*k*_+*y*_*k*_>50, *x*_*k*_/*y*_*k*_<10 and *y*_*k*_/*x*_*k*_<10. The criteria made it very unlikely to observe biallelic expression because of sequencing error. For each SNP that met the criteria in *n* samples, if no allele was preferably expressed by the transcription machinery, *z* should follow a binomial distribution *f*(*z*; *n*, *p*) with *p*=0.5. To obtain enough testing power, SNPs with *n* ≥ 8 were deemed qualified for the binomial test. Under the above criteria, we found 24 qualified SNPs and listed all 20 significant testing results in Table 5.

## Competing interests

The authors declare that they have no competing interests.

## Authors’ contributions

HL and SL developed the model of dsPIG. HL carried out the analysis. XS built the R package. JG built the website. YL performed sequence alignment. YJ improved the model. HL and SL wrote the manuscript. SL conceived and directed this study. JJM co-directed this study. All authors read and approved the final manuscript.

## Supplementary Material

Additional file 1 **Figure S1.** Distribution of simulation-generated allelic counts vs. observed distribution in real data. Red line stands for the generated distribution; black line stands for the observed distribution. The red text and black text in the upper right green box are summarized statistics for red line and black line, respectively Click here for file

Additional file 2 **Figure S2.** Flowchart showing steps in data simulation and model assessment. In step 2, the differences in data generation are caused by two factors: (*i*) imprinted genes need to express only one allele at a tissue level while non-imprinted genes don’t, (*ii*) two alleles expressed from non-imprinted genes need to be sequenced in RNA-Seq with an equal probability, while imprinted genes only have one allele expressed. In this step we also need to assume that sequencing error leads to misread of one nucleotide to the other three with an equal probability. RT-PCR amplification is not shown in the process because we assume that it amplifies both alleles synchronously (for details, see Discussion) Click here for file

Additional file 3 **Table S1.** The predicted imprinted genes based on mRNA-Seq data from Group I and Group II samples. Abbreviations: rs#-SNP identification number, Chr-chromosome, Str-strand, SS-sample size. “NA” in the “FDR” column means the FDR could not be estimated based on our 20,000-time simulations Click here for file

Additional file 4 **Table S2.**The FDR values with respect to different sample sizes and allele frequencies. “ NA” means FDR could not be estimated based on our 20,000-time simulations Click here for file

Additional file 5 **Figure S3.** Distribution of SNPs’ sample sizes in Group I (from 9 diverse tissue samples) and Group II (from 20 cerebellum samples). Group I and Group II had a total of 44007 SNPs and 66294 SNPs with sample size >0, respectively Click here for file

Additional file 6 **Figure S4.** Functional enrichment analysis of the 57 genes and the 94 genes in Ingenuity® Pathway Analysis. (*a*) Comparison of enrichment in Bio Functions between the two gene lists. (*b*) Comparison of enrichment in Canonical Pathways between the two gene lists. The height of each bar in (*a*) and (*b*) represents the logartihm (10-based) transformed p-values calculated from Fisher’s exact test. In (*a*), the horizontal yellow line is the threshold [i.e., -log_10_(0.05)] above which bars (p-values) were considered significant; in (*b*), there is no bar above the threshold which is not shown here. (Figure S4 is located in a separate PDF file: “Fig. S4.pdf”.) Click here for file

Additional file 7 **Figure S5.** Sensitivity and specificity analysis of dsPIG based on the genes with known patterns of allelic expression. (*a*) Sensitivity analysis based on the validated imprinted genes. (*b*) Specificity analysis based on the validated non-imprinted genes. In (*a*) and (*b*), the solid black lines, which showed the numbers of genes identified by dsPIG as “imprinted”, were based on the mRNA-Seq data of Group I samples, and the dotted black lines were based on Group II samples; the red line is the cut-off (0.2) used in this study to predict imprinted genes. (*c*) ROC curve for dsPIG based on Group I samples. (*d*) ROC curve for dsPIG based on Group II samples Click here for file

Additional file 8 R package (dsPIG, version 3.0) for UNIX). Click here for file

Additional file 9 R package (dsPIG, version 3.0) for Windows). Click here for file

Additional file 10 The instruction and the sample files for the R package of dsPIG. Click here for file

Additional file 11 The annotated R code and C code for dsPIG used in our study. Click here for file

Additional file 12 **Figure S6.** Simulated (log-transformed) posteriors of genes with biallelic expression in only one sample. Each positive integer (*x*) on the *x*-axis (“Sample size”) includes two parts: 1 sample of biallelic expression and (*x*-1) samples of imprinted expression. Posteriors were calculated by dsPIG. The dashed line stands for the log-transformed prior (0.01). This result was based on 20,000-time simulations with geometric mean as the method of averaging posteriors Click here for file

Additional file 13 **Figure S7.** Distributions of QS in 32 samples (including 3 breast cancer cell line samples). The *x*-axis is the number of sequencing tags that covered the SNP site, and the *y*-axis is the QS. Tissue names are located at the lower right side of each plot, where “Cancer” stands for “breast cancer cell line sample” and “C.” stands for “cerebellum sample”. Dashed lines in the 32 samples represent the cut-off (0.9) for QS Click here for file

## References

[B1] Gimelbrant AA Hutchinson JN Thompson BR Chess A Widespread monoallelic expression on human autosomes Science 2007 318 1136 1140 10.1126/science.1148910 18006746

[B2] Lyon MF X chromosomes and dosage compensation Nature 1986 320 313 3960115 10.1038/320313b0

[B3] Pernis B Chiappino G Kelus AS Gell PG Cellular localization of immunoglobulins with different allotypic specificities in rabbit lymphoid tissues J Exp Med 1965 122 853 876 10.1084/jem.122.5.853 4159057 PMC2138120

[B4] Chess A Simon I Cedar H Axel R Allelic inactivation regulates olfactory receptor gene expression Cell 1994 78 823 834 10.1016/S0092-8674(94)90562-2 8087849

[B5] Rajewsky K Clonal selection and learning in the antibody system Nature 1996 381 751 758 10.1038/381751a0 8657279

[B6] Hollander GA Zuklys S Morel C Mizoguchi E Mobisson K Simpson S Terhorst C Wishart W Golan DE Bhan AK Burakoff SJ Monoallelic expression of the interleukin-2 locus Science 1998 279 2118 2121 10.1126/science.279.5359.2118 9516115

[B7] Bix M Locksley RM Independent and epigenetic regulation of the interleukin-4 alleles in CD4+ T Cells Science 1998 281 1352 1354 9721100 10.1126/science.281.5381.1352

[B8] Rhoades KL Singh N Simon I Glidden B Cedar H Chess A Allele-specific expression patterns of interleukin-2 and Pax-5 revealed by a sensitive single-cell RT-PCR analysis Curr Biol 2000 10 789 792 10.1016/S0960-9822(00)00565-0 10898982

[B9] Gimelbrant AA Ensminger AW Qi P Zucher J Chess A Monoallelic Expression and Asynchronous Replication of p120 Catenin in Mouse and Human Cells J Biol Chem 2005 280 1354 1359 15522875 10.1074/jbc.M411283200

[B10] Reik W Walter J Genomic imprinting: parental influence on the genome Nat Rev Genet 2001 2 21 32 11253064 10.1038/35047554

[B11] Ferguson-Smith AC Surani MA Imprinting and the epigenetic asymmetry between parental genomes Science 2001 293 1086 1089 10.1126/science.1064020 11498578

[B12] Morison IM Ramsay JP Spencer HG A census of mammalian imprinting Trends Genet 2005 21 457 465 10.1016/j.tig.2005.06.008 15990197

[B13] Zhang TY Meaney MJ Epigenetics and the environmental regulation of the genome and its function Annu Rev Psychol 2010 61 439 466 10.1146/annurev.psych.60.110707.163625 19958180

[B14] Constância M Pickard B Kelsey G Reik W Imprinting mechanisms Genome Res 1998 8 881 900 9750189 10.1101/gr.8.9.881

[B15] Tycko B Morison IM Physiological functions of imprinted genes J Cell Physiol 2002 192 245 258 10.1002/jcp.10129 12124770

[B16] Isles AR Holland AJ Imprinted genes and mother-offspring interactions Early Hum Dev 2005 81 73 77 10.1016/j.earlhumdev.2004.10.006 15707717

[B17] Caron H van Sluis P van Hoeve M de Kraker J Bras J Slater R Mannens M Voute PA Westerveld A Versteeg R Allelic loss of chromosome 1p36 in neuroblastoma is preferential maternal origin and correlates with N-myc amplification Nat Genet 1993 4 187 190 10.1038/ng0693-187 8102298

[B18] Moulton T Chung WY Yuan L Hensle T Waber P Nisen P Tycko B Genomic imprinting in Wilms’ tumor Med Ped Oncol 1996 27 476 483 10.1002/(SICI)1096-911X(199611)27:5<476::AID-MPO15>3.0.CO;2-8 8827077

[B19] Bartolomei MS Tilghman SM Genomic imprinting in mammals Annu Rev Genet 1997 32 493 525 9442905 10.1146/annurev.genet.31.1.493

[B20] Nicholls RD Saitoh S Horsthemke B Imprinting in Prader-willi and Angelman syndromes Trends Genet 1998 14 194 200 10.1016/S0168-9525(98)01432-2 9613204

[B21] Waterland RA Jirtle RL Early nutrition, epigenetic changes at transposons and imprinted genes, and enhanced susceptibility to adult chronic diseases Nutrition 2004 20 63 68 10.1016/j.nut.2003.09.011 14698016

[B22] Jirtle RL Skinner MK Environmental epigenomics and disease susceptibility Nat Rev Genet 2007 8 253 262 10.1038/nrg2045 17363974 PMC5940010

[B23] Falls JG Pulford DJ Wylie AA Jirtle RL Genomic imprinting: implications for human disease Am J Pathol 1999 154 635 647 10.1016/S0002-9440(10)65309-6 10079240 PMC1866410

[B24] Murphy SK Jirtle RL Imprinting evolution and the price of silence Bioessays 2003 25 577 588 10.1002/bies.10277 12766947

[B25] Wilkinson LS Davies W Isles AR Genomic imprinting effects on brain development and function Nat Rev Neurosci 2007 8 832 843 10.1038/nrn2235 17925812

[B26] Nikaido I Saito C Mizuno Y Meguro M Bono H Kadomura M Kono T Morris GA Lyons PA Oshimura M Discovery of imprinted transcripts in the mouse transcriptome using large-scale expression profiling Genome Res 2003 13 1402 1409 10.1101/gr.1055303 12819139 PMC403673

[B27] Lo HS Wang Z Hu Y Yang HH Gere S Buetow KH Lee MP Allelic variation in gene expression is common in the human genome Genome Res 2003 13 1855 1862 12902379 10.1101/gr.1006603 PMC403776

[B28] Luedi PP Hartemink AJ Jirtle RL Genome-wide prediction of imprinted murine genes Genome Res 2005 15 875 884 10.1101/gr.3303505 15930497 PMC1142478

[B29] Pant PV Tao H Beilharz EJ Ballinger DG Cox DR Frazer KA Analysis of allelic differential expression in human white blood cells Genome Res 2006 16 331 339 10.1101/gr.4559106 16467561 PMC1415206

[B30] Ruf N Dunzinger U Brinckmann A Haaf T Nurnberg P Zechner U Expression profiling of uniparental mouse embryos is inefficient in identifying novel imprinted genes Genomics 2006 87 509 519 10.1016/j.ygeno.2005.12.007 16455231

[B31] Luedi PP Dietrich FS Weidman JR Bosko JM Jirtle RL Hartemink AJ Computational and experimental identification of novel human imprinted genes Genome Res 2007 17 1723 1730 10.1101/gr.6584707 18055845 PMC2099581

[B32] Mardis ER The impact of next generation sequencing technology on genetics Trends Genet 2008 24 133 141 10.1016/j.tig.2007.12.007 18262675 PMC2680276

[B33] Park PJ ChIP-seq: advantages and challenges of a maturing technology Nat Rev Genet 2009 10 669 680 19736561 10.1038/nrg2641 PMC3191340

[B34] Wang Z Gerstein M Snyder M RNA-Seq: a revolutionary tool for transcriptomics Nat Rev Genet 2009 10 57 63 10.1038/nrg2484 19015660 PMC2949280

[B35] Babak T DeVeale B Armour C Raymond C Cleary MA van der Kooy D Johnson JM Lim LP Global survey of genomic imprinting by transcriptome sequencing Curr Biol 2008 18 1735 1741 10.1016/j.cub.2008.09.044 19026546

[B36] Watanabe DB Barlow DP Random and imprinted monoallelic expression Genes Cells 1996 1 795 802 10.1046/j.1365-2443.1996.d01-276.x 9077434

[B37] Sherry ST Ward MH Kholodov M Baker J Phan L Smigielski EM Sirotkin K: dbSNP: the NCBI database of genetic variation Nucleic Acids Res 2001 29 308 311 10.1093/nar/29.1.308 11125122 PMC29783

[B38] Wang ET Sandberg R Luo S Khrebtukova I Zhang L Mayr C Kingsmore SF Schroth GP Burge CB Alternative isoform regulation in human tissue transcriptomes Nature 2008 456 470 476 10.1038/nature07509 18978772 PMC2593745

[B39] Mudge J Miller NA Khrebtukova I Lindquist IE May GD Huntley JJ Luo S Zhang L van Velkinburgh JC Farmer AD Genomic Convergence Analysis of Schizophrenia: mRNA sequencing reveals altered synaptic vesicular transport in post-mortem cerebellum PLoS One 2008 3 e3625 10.1371/journal.pone.0003625 18985160 PMC2576459

[B40] Benjamini Y Hochberg Y Controlling the false discovery rate: a practical and powerful approach to multiple testing J. R. Stat. Soc. B 1995 57 289 300

[B41] Morison IM Paton CJ Cleverley SD The imprinted gene and parent-of-origin effect database Nucleic Acids Res 2001 29 275 276 10.1093/nar/29.1.275 11125110 PMC29803

[B42] Lewis A Reik W How imprinting centres work Cytogenet Genome Res 2006 113 1 4 10.1159/000090818 16575166

[B43] Willing MC Deschenes SP Slayton RL Roberts EJ Premature chain termination is a unifying mechanism for COL1A1 null alleles in osteogenesis imperfecta type I cell strains Am J Hum Genet 1996 59 799 809 8808594 PMC1914787

[B44] Yan H Yuan W Velculescu VE Vogelstein B Kinzler KW Allelic variation in human gene expression Science 2002 297 1143 10.1126/science.1072545 12183620

[B45] Pastinen T Sladek R Gurd S Sammak A Ge B Lepage P Lavergne K Villeneuve A Gaudin T Brandstrom H A survey of genetic and epigenetic variation affecting human gene expression Physiol Genomics 2004 16 184 193 14583597 10.1152/physiolgenomics.00163.2003

[B46] Pastinen T Hudson TJ Cis-acting regulatory variation in the human genome Science 2004 306 647 10.1126/science.1101659 15499010

[B47] Karolchik D Hinrichs AS Furey TS Roskin KM Sugnet CW Haussler D Kent WJ The UCSC Table Browser data retrieval tool Nucleic Acids Res 2004 32 D493 D496 10.1093/nar/gkh103 14681465 PMC308837

