# Supplementary figures and images for: dsPIG: a tool to predict imprinted genes from the deep sequencing of whole transcriptomes
Source: BMC Bioinformatics. 2012 Oct 19;13:271. doi: 10.1186/1471-2105-13-271 (PMC3497615; doi:10.1186/1471-2105-13-271)

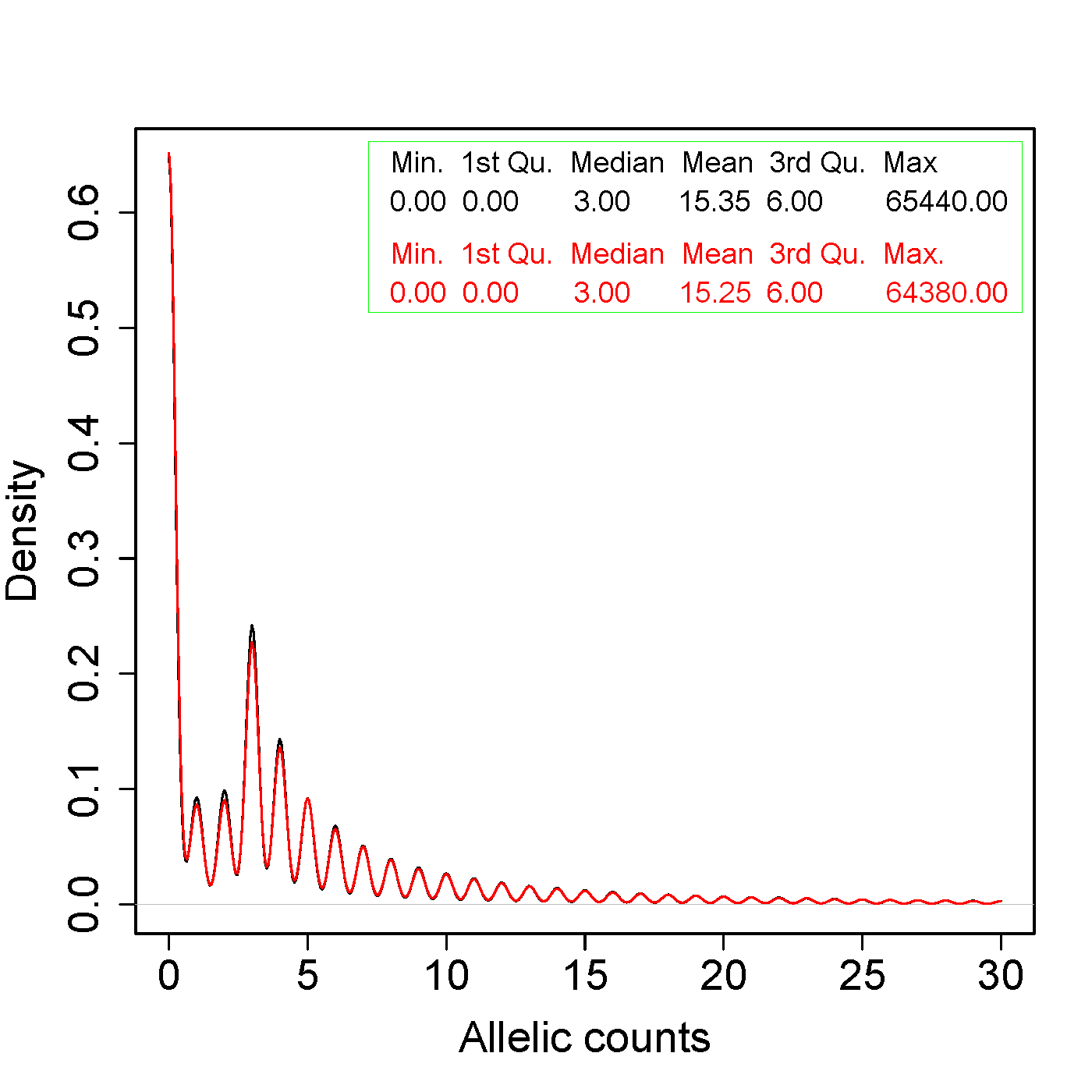

Supplement: Additional file 1 — Figure S1. Distribution of simulation-generated allelic counts vs. observed distribution in real data. Red line stands for the generated distribution; black line stands for the observed distribution. The red text and black text in the upper right green box are summarized statistics for red line and black line, respectively [file 1471-2105-13-271-S1.tiff]

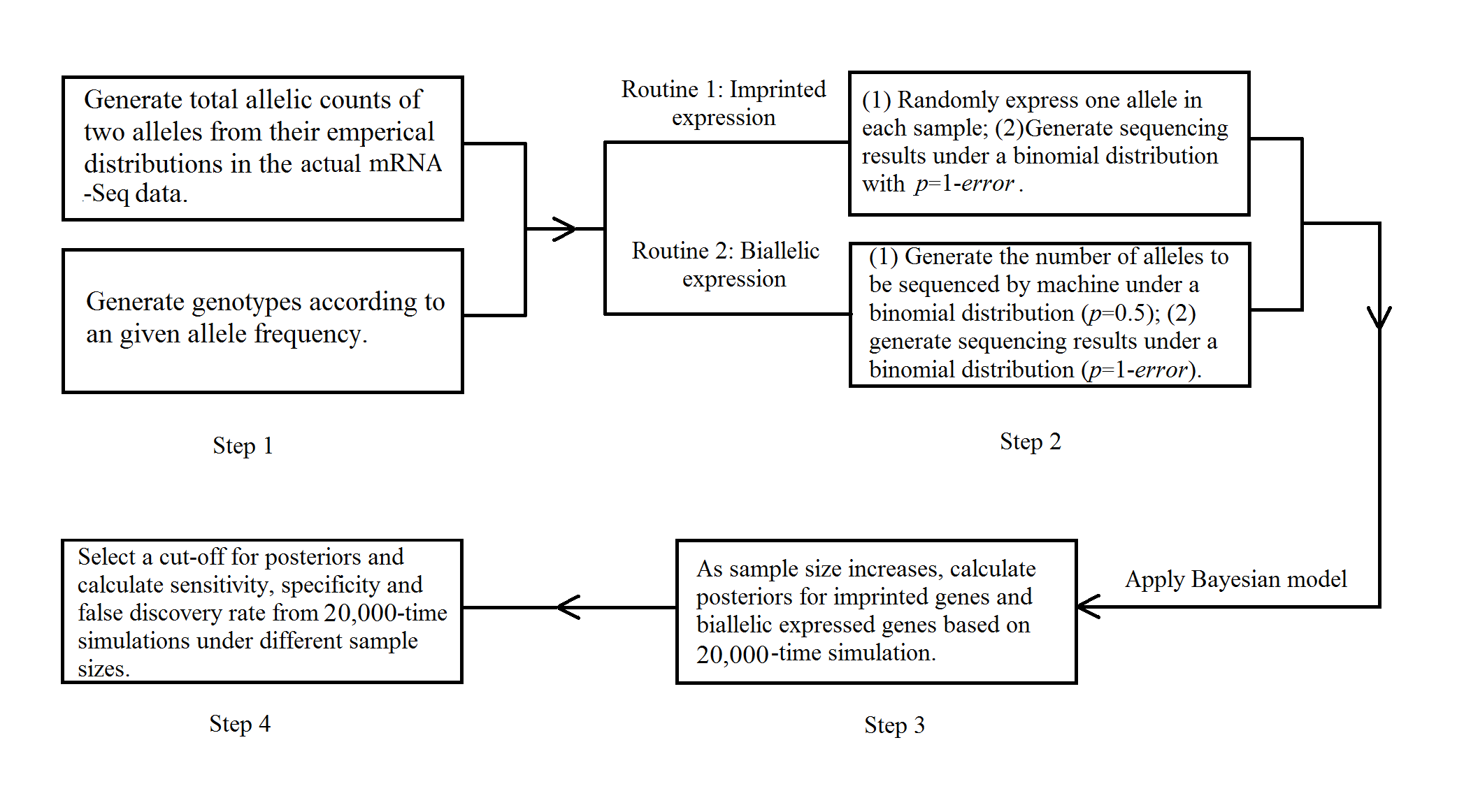

Supplement: Additional file 2 — Figure S2. Flowchart showing steps in data simulation and model assessment. In step 2, the differences in data generation are caused by two factors: (i) imprinted genes need to express only one allele at a tissue level while non-imprinted genes don’t, (ii) two alleles expressed from non-imprinted genes need to be sequenced in RNA-Seq with an equal probability, while imprinted genes only have one allele expressed. In this step we also need to assume that sequencing error leads to misread of one nucleotide to the other three with an equal probability. RT-PCR amplification is not shown in the process because we assume that it amplifies both alleles synchronously (for details, see Discussion) [file 1471-2105-13-271-S2.tiff]

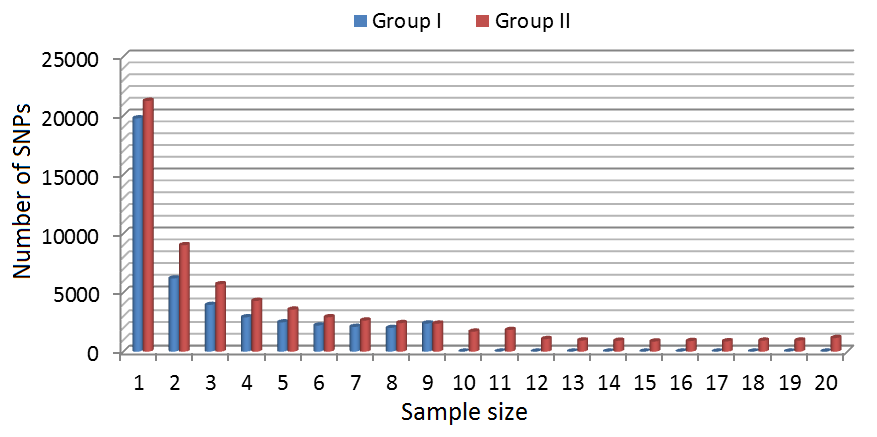

Supplement: Additional file 5 — Figure S3. Distribution of SNPs’ sample sizes in Group I (from 9 diverse tissue samples) and Group II (from 20 cerebellum samples). Group I and Group II had a total of 44007 SNPs and 66294 SNPs with sample size >0, respectively [file 1471-2105-13-271-S5.png]

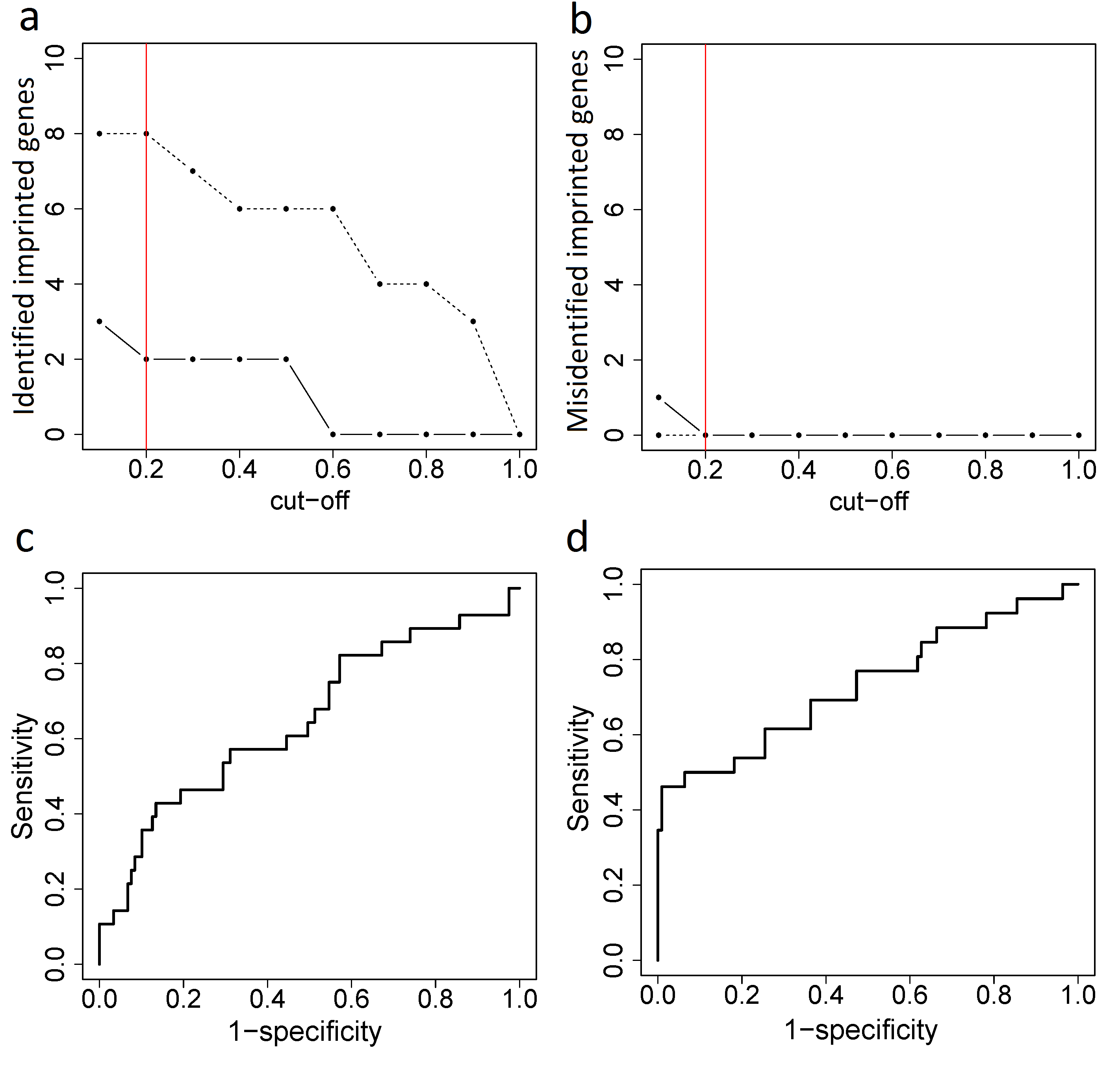

Supplement: Additional file 7 — Figure S5. Sensitivity and specificity analysis of dsPIG based on the genes with known patterns of allelic expression. (a) Sensitivity analysis based on the validated imprinted genes. (b) Specificity analysis based on the validated non-imprinted genes. In (a) and (b), the solid black lines, which showed the numbers of genes identified by dsPIG as “imprinted”, were based on the mRNA-Seq data of Group I samples, and the dotted black lines were based on Group II samples; the red line is the cut-off (0.2) used in this study to predict imprinted genes. (c) ROC curve for dsPIG based on Group I samples. (d) ROC curve for dsPIG based on Group II samples [file 1471-2105-13-271-S7.png]

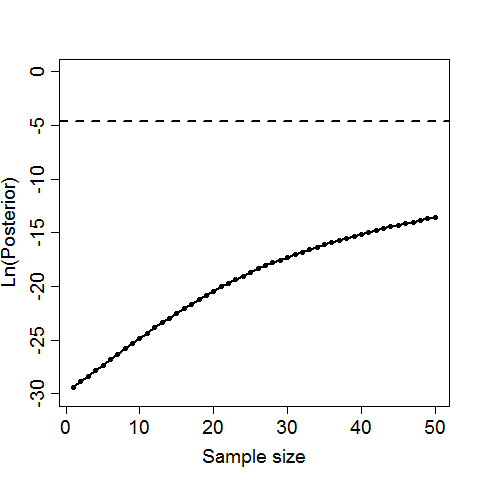

Supplement: Additional file 12 — Figure S6. Simulated (log-transformed) posteriors of genes with biallelic expression in only one sample. Each positive integer (x) on the x-axis (“Sample size”) includes two parts: 1 sample of biallelic expression and (x-1) samples of imprinted expression. Posteriors were calculated by dsPIG. The dashed line stands for the log-transformed prior (0.01). This result was based on 20,000-time simulations with geometric mean as the method of averaging posteriors [file 1471-2105-13-271-S12.png]

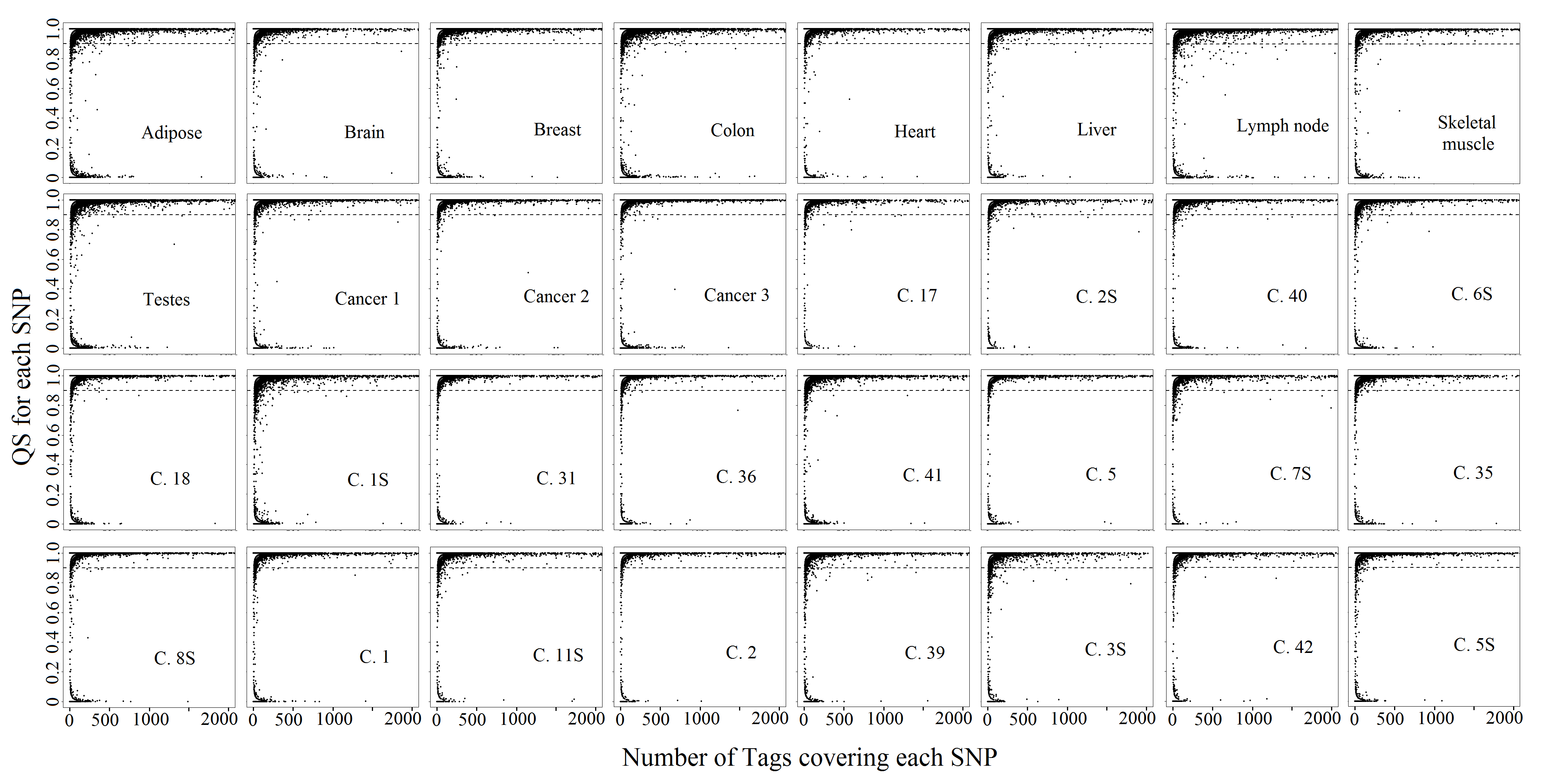

Supplement: Additional file 13 — Figure S7. Distributions of QS in 32 samples (including 3 breast cancer cell line samples). The x-axis is the number of sequencing tags that covered the SNP site, and the y-axis is the QS. Tissue names are located at the lower right side of each plot, where “Cancer” stands for “breast cancer cell line sample” and “C.” stands for “cerebellum sample”. Dashed lines in the 32 samples represent the cut-off (0.9) for QS [file 1471-2105-13-271-S13.png]
