# Supplementary material for: dsPIG: a tool to predict imprinted genes from the deep sequencing of whole transcriptomes
Source: BMC Bioinformatics. 2012 Oct 19;13:271. doi: 10.1186/1471-2105-13-271 (PMC3497615; doi:10.1186/1471-2105-13-271)
Supplement: Additional file 3 — Table S1. The predicted imprinted genes based on mRNA-Seq data from Group I and Group II samples. Abbreviations: rs#-SNP identification number, Chr-chromosome, Str-strand, SS-sample size. “NA” in the “FDR” column means the FDR could not be estimated based on our 20,000-time simulations [file 1471-2105-13-271-S3.doc]

**Table S1 The predicted imprinted genes based on mRNA-Seq data from Group I and Group II samples.** Abbreviations: rs#-SNP identification number, Chr-chromosome, Str-strand, SS-sample size. “NA” in the “FDR” column means the FDR could not be estimated based on our 20,000-time simulations.

| **SNP(rs#)** | **Chr** | **Location** | **Str** | **Posterior** | **Gene ID** | **Gene Symbol** | **SS** | **FDR** | **Group** |
| --- | --- | --- | --- | --- | --- | --- | --- | --- | --- |
| **11538691** | chr17 | 4789783 | + | 1 | 5216 | PFN1 | 9 | 0 | I |
| **178412** | chr7 | 73173272 | - | 1 | 3984 | LIMK1 | 8 | 0 | I |
| **17094371** | chr14 | 57677831 | + | 1 | 145407 | C14orf37 | 9 | 0 | I |
| **2596331** | chr1 | 1.44E+08 | - | 0.999996 | 9554 | SEC22B | 7 | 0.26 | I |
| **8110904** | chr19 | 47723208 | + | 0.999995 | 634 | CEACAM1 | 4 | 0 | I |
| **11555395** | chr17 | 67629054 | + | 0.999993 | 6662 | SOX9 | 5 | 0 | I |
| **4015375** | chr7 | 89628110 | + | 0.99997 | 26872 | STEAP1 | 6 | 0 | I |
| **4015375** | chr7 | 89628110 | + | 0.99997 | 256227 | MGC87042 | 6 | 0 | I |
| **10208923** | chr2 | 1.41E+08 | + | 0.9998 | 53353 | LRP1B | 5 | 0 | I |
| **584959** | chr3 | 61703763 | + | 0.995669 | 1E+08 | RPL10AP6 | 9 | 0.052 | I |
| **10800864** | chr1 | 2.01E+08 | + | 0.995398 | 10765 | KDM5B | 9 | 0.052 | I |
| **10306** | chr10 | 74437407 | - | 0.993645 | 5033 | P4HA1 | 4 | 0 | I |
| **2009646** | chr5 | 1.08E+08 | + | 0.989758 | 2241 | FER | 6 | 0 | I |
| **2722863** | chr9 | 1.2E+08 | - | 0.985673 | 389787 | LOC389787 | 4 | 0 | I |
| **7928246** | chr11 | 1.07E+08 | + | 0.973873 | 55531 | ELMOD1 | 4 | 0 | I |
| **1736924** | chr6 | 29800989 | - | 0.965236 | 3134 | HLA-F | 4 | 0 | I |
| **3827521** | chr12 | 93467186 | - | 0.908228 | 283398 | LOC283398 | 7 | 0 | I |
| **12217419** | chr10 | 31824721 | + | 0.896346 | 6935 | ZEB1 | 4 | 0 | I |
| **12695** | chr4 | 6695173 | + | 0.860493 | 93621 | MRFAP1 | 9 | 0 | I |
| **7179** | chr16 | 14897344 | + | 0.834212 | 283820 | NOMO2 | 4 | 0.629 | I |
| **2291063** | chr1 | 32485785 | - | 0.776045 | 84734 | FAM167B | 2 | NA | I |
| **11953084** | chr5 | 1.77E+08 | + | 0.704415 | 653314 | RPL19P9 | 7 | NA | I |
| **6777333** | chr3 | 44596329 | + | 0.640663 | 55888 | ZNF167 | 4 | NA | I |
| **8075077** | chr17 | 15548918 | + | 0.56027 | 57335 | ZNF286A | 6 | 0.671 | I |
| **4652** | chr14 | 54674788 | + | 0.5037 | 3958 | LGALS3 | 7 | 0.479 | I |
| **3971706** | chr19 | 61697880 | + | 0.478128 | 1E+08 | LOC100128252 | 4 | 0.343 | I |
| **2000203** | chr6 | 73753786 | - | 0.467888 | 56479 | KCNQ5 | 5 | 0.517 | I |
| **10119** | chr19 | 50098512 | - | 0.464521 | 10452 | TOMM40 | 8 | 0.373 | I |
| **503068** | chr6 | 1.08E+08 | - | 0.444198 | 11231 | SEC63 | 3 | 0.679 | I |
| **1572611** | chr14 | 54903626 | - | 0.431973 | 22863 | KIAA0831 | 7 | 0.565 | I |
| **2018711** | chr2 | 1.1E+08 | - | 0.431052 | 4867 | NPHP1 | 4 | 0.698 | I |
| **7410** | chr14 | 67214175 | + | 0.408688 | 51109 | RDH11 | 8 | 0.444 | I |
| **11541040** | chr12 | 74728086 | + | 0.399748 | 4673 | NAP1L1 | 3 | 0.679 | I |
| **4754** | chr4 | 89121715 | + | 0.38997 | 6696 | SPP1 | 7 | 0.412 | I |
| **1043165** | chr1 | 1.54E+08 | - | 0.35134 | 23381 | SMG5 | 7 | 0.565 | I |
| **4644** | chr14 | 54674687 | + | 0.340277 | 3958 | LGALS3 | 6 | 0.696 | I |
| **2014576** | chr19 | 50960915 | - | 0.314464 | 147912 | SIX5 | 6 | 0.696 | I |
| **2014576** | chr19 | 50960915 | - | 0.314464 | 388553 | LOC388553 | 6 | 0.696 | I |
| **6503513** | chr17 | 34815138 | + | 0.309561 | 5469 | MED1 | 8 | 0.556 | I |
| **1045216** | chr10 | 1.24E+08 | + | 0.285049 | 59338 | PLEKHA1 | 8 | 0.516 | I |
| **2627788** | chr8 | 1.08E+08 | + | 0.276622 | 55074 | OXR1 | 8 | 0.516 | I |
| **1127155** | chr7 | 72884396 | + | 0.2707 | 1364 | CLDN4 | 4 | 0.594 | I |
| **2078348** | chr1 | 28435670 | + | 0.258369 | 93974 | ATPIF1 | 5 | 0.59 | I |
| **1979572** | chr17 | 25536103 | - | 0.25299 | 84081 | CCDC55 | 7 | 0.587 | I |
| **216463** | chr17 | 25877655 | + | 0.246612 | 9527 | GOSR1 | 9 | 0.535 | I |
| **1681904** | chr8 | 1.08E+08 | + | 0.24614 | 55074 | OXR1 | 6 | 0.719 | I |
| **1129156** | chr19 | 45410915 | + | 0.244727 | 4294 | MAP3K10 | 8 | 0.628 | I |
| **4399146** | chr1 | 1.55E+08 | + | 0.235202 | 3068 | HDGF | 9 | 0.579 | I |
| **9299214** | chr9 | 1.15E+08 | + | 0.233495 | 246184 | CDC26 | 8 | 0.572 | I |
| **2294226** | chr1 | 26019035 | - | 0.230406 | 646471 | LOC646471 | 6 | 0.673 | I |
| **1611772** | chr1 | 1.53E+08 | - | 0.230346 | 4582 | MUC1 | 3 | 0.636 | I |
| **1127156** | chr7 | 72884431 | + | 0.227674 | 1364 | CLDN4 | 3 | 0.735 | I |
| **11316** | chr7 | 72884490 | + | 0.227347 | 1364 | CLDN4 | 3 | 0.735 | I |
| **9392** | chr16 | 3715169 | + | 0.225266 | 1387 | CREBBP | 6 | 0.696 | I |
| **1471089** | chr1 | 2.38E+08 | - | 0.222455 | 56776 | FMN2 | 3 | 0.735 | I |
| **2715860** | chr2 | 9445986 | + | 0.221908 | 8853 | ASAP2 | 5 | 0.751 | I |
| **8165** | chr2 | 2.32E+08 | - | 0.217124 | 5147 | PDE6D | 6 | 0.673 | I |
| **6942** | chr2 | 1.9E+08 | + | 0.212629 | 94101 | ORMDL1 | 5 | 0.725 | I |
| **10988** | chr5 | 1.45E+08 | + | 0.209055 | 51520 | LARS | 9 | 0.579 | I |
| **2305059** | chr19 | 6370274 | + | 0.206468 | 8570 | KHSRP | 6 | 0.696 | I |
| **2939635** | chr8 | 56527146 | + | 0.205567 | 114786 | XKR4 | 7 | 0.647 | I |
| **178412** | chr7 | 73173272 | - | 1 | 3984 | LIMK1 | 11 | 0.098 | II |
| **11538691** | chr17 | 4789783 | + | 1 | 5216 | PFN1 | 20 | 0.023 | II |
| **11541557** | chr1 | 2.26E+08 | + | 1 | 375 | ARF1 | 9 | 0 | II |
| **17492855** | chr2 | 1.59E+08 | + | 0.999999 | 130940 | CCDC148 | 18 | 0.014 | II |
| **2352731** | chr3 | 1.44E+08 | + | 0.999995 | 5089 | PBX2 | 10 | 0.072 | II |
| **1065453** | chr7 | 99755171 | - | 0.999992 | 441272 | SPDYE3 | 5 | 0 | II |
| **11066116** | chr12 | 1.11E+08 | + | 0.999989 | 89894 | TMEM116 | 8 | 0 | II |
| **2499** | chr6 | 30021520 | - | 0.999982 | 3105 | HLA-A | 7 | 0.26 | II |
| **3093976** | chr6 | 31610839 | - | 0.99993 | 7919 | BAT1 | 8 | 0.12 | II |
| **3132453** | chr6 | 31712022 | - | 0.999913 | 7916 | BAT2 | 4 | 0 | II |
| **1051470** | chr12 | 1.17E+08 | + | 0.999874 | 5037 | PEBP1 | 14 | 0.051 | II |
| **3131628** | chr6 | 31610745 | - | 0.999788 | 7919 | BAT1 | 7 | 0 | II |
| **584959** | chr3 | 61703763 | + | 0.999517 | 1E+08 | RPL10AP6 | 11 | 0.055 | II |
| **1129640** | chr6 | 31614602 | - | 0.999417 | 7919 | BAT1 | 7 | 0 | II |
| **16962951** | chr17 | 21259999 | + | 0.998954 | 3768 | KCNJ12 | 11 | 0.055 | II |
| **1043483** | chr6 | 31001706 | + | 0.998608 | 57176 | VARS2 | 14 | 0.051 | II |
| **3093948** | chr6 | 31609391 | - | 0.998283 | 7919 | BAT1 | 7 | 0 | II |
| **2272593** | chr6 | 31709322 | - | 0.998224 | 7916 | BAT2 | 9 | 0.052 | II |
| **1055388** | chr6 | 31609715 | - | 0.996835 | 7919 | BAT1 | 7 | 0 | II |
| **1059288** | chr6 | 33375649 | - | 0.99527 | 5863 | RGL2 | 13 | 0.057 | II |
| **1059288** | chr6 | 33375649 | - | 0.99527 | 6892 | TAPBP | 13 | 0.057 | II |
| **7928246** | chr11 | 1.07E+08 | + | 0.99497 | 55531 | ELMOD1 | 4 | 0 | II |
| **2000203** | chr6 | 73753786 | - | 0.993102 | 56479 | KCNQ5 | 13 | 0.069 | II |
| **1046080** | chr6 | 31703860 | + | 0.992397 | 7916 | BAT2 | 8 | 0 | II |
| **1266076** | chr6 | 31748496 | - | 0.987191 | 58496 | LY6G5B | 16 | 0.028 | II |
| **7179** | chr16 | 14897344 | + | 0.979473 | 283820 | NOMO2 | 7 | 0 | II |
| **1475865** | chr6 | 31765391 | - | 0.979016 | 7920 | BAT5 | 7 | 0 | II |
| **6531** | chr6 | 33271428 | - | 0.977748 | 6257 | RXRB | 6 | 0 | II |
| **11555395** | chr17 | 67629054 | + | 0.975199 | 6662 | SOX9 | 2 | NA | II |
| **1061783** | chr6 | 33390605 | - | 0.968048 | 6892 | TAPBP | 12 | 0.076 | II |
| **1061783** | chr6 | 33390605 | - | 0.968048 | 9278 | ZBTB22 | 12 | 0.076 | II |
| **2009646** | chr5 | 1.08E+08 | + | 0.962144 | 2241 | FER | 6 | 0 | II |
| **1046089** | chr6 | 31710945 | + | 0.959646 | 7916 | BAT2 | 16 | 0.035 | II |
| **3106189** | chr6 | 33389979 | - | 0.952453 | 6892 | TAPBP | 12 | 0.076 | II |
| **3106189** | chr6 | 33389979 | - | 0.952453 | 9278 | ZBTB22 | 12 | 0.076 | II |
| **2071888** | chr6 | 33380832 | - | 0.931622 | 6892 | TAPBP | 10 | 0.146 | II |
| **3130250** | chr6 | 29732979 | + | 0.918532 | 4340 | MOG | 4 | 0 | II |
| **14115** | chr22 | 28493525 | + | 0.889141 | 29796 | UCRC | 15 | 0.039 | II |
| **14115** | chr22 | 28493525 | + | 0.889141 | 55954 | ZMAT5 | 15 | 0.039 | II |
| **8075077** | chr17 | 15548918 | + | 0.872835 | 57335 | ZNF286A | 9 | 0.211 | II |
| **4985866** | chr17 | 21259991 | + | 0.858971 | 3768 | KCNJ12 | 11 | 0.055 | II |
| **10636** | chr16 | 55200843 | + | 0.709334 | 4502 | MT2A | 10 | 0.142 | II |
| **2018711** | chr2 | 1.1E+08 | - | 0.701176 | 4867 | NPHP1 | 6 | 0.309 | II |
| **526897** | chr2 | 2.19E+08 | + | 0.683651 | 9125 | RQCD1 | 13 | 0.128 | II |
| **6963** | chr17 | 37985122 | - | 0.661404 | 29893 | PSMC3IP | 12 | 0.146 | II |
| **10897471** | chr11 | 63530945 | + | 0.631345 | 28992 | MACROD1 | 8 | 0.233 | II |
| **879027** | chr14 | 76562643 | + | 0.626486 | 64207 | C14orf4 | 12 | 0.201 | II |
| **2744537** | chr6 | 33270192 | - | 0.598436 | 1302 | COL11A2 | 4 | 0 | II |
| **2744537** | chr6 | 33270192 | - | 0.598436 | 6257 | RXRB | 4 | 0 | II |
| **420599** | chr3 | 11863258 | - | 0.52856 | 132001 | C3orf31 | 8 | 0.346 | II |
| **420599** | chr3 | 11863258 | - | 0.52856 | 1E+08 | LOC100129929 | 8 | 0.346 | II |
| **506008** | chr1 | 1.1E+08 | - | 0.503053 | 2948 | GSTM4 | 3 | 0.466 | II |
| **690124** | chr17 | 70385846 | - | 0.497359 | 283985 | FADS6 | 16 | 0.301 | II |
| **10782959** | chr1 | 93584156 | + | 0.488268 | 1810 | DR1 | 9 | 0.33 | II |
| **10782959** | chr1 | 93584156 | + | 0.488268 | 1E+08 | LOC100131564 | 9 | 0.33 | II |
| **1028564** | chr11 | 33118584 | - | 0.4792 | 1479 | CSTF3 | 8 | 0.373 | II |
| **2764657** | chr1 | 76938089 | - | 0.447453 | 729708 | RCTPI1 | 3 | 0.381 | II |
| **1043618** | chr6 | 31891485 | + | 0.441358 | 3303 | HSPA1A | 11 | 0.389 | II |
| **1043618** | chr6 | 31891485 | + | 0.441358 | 3305 | HSPA1L | 11 | 0.389 | II |
| **2295963** | chr9 | 4654851 | + | 0.418156 | 403313 | PPAPDC2 | 15 | 0.318 | II |
| **3679** | chr19 | 7884429 | - | 0.408834 | 5609 | MAP2K7 | 9 | 0.432 | II |
| **3130100** | chr6 | 33391743 | + | 0.407145 | 6892 | TAPBP | 7 | 0.565 | II |
| **3130100** | chr6 | 33391743 | + | 0.407145 | 9278 | ZBTB22 | 7 | 0.565 | II |
| **1130641** | chr12 | 49923507 | + | 0.406466 | 9802 | DAZAP2 | 12 | 0.298 | II |
| **2295962** | chr9 | 4654835 | + | 0.393253 | 55064 | C9orf68 | 9 | 0.432 | II |
| **2295962** | chr9 | 4654835 | + | 0.393253 | 403313 | PPAPDC2 | 9 | 0.432 | II |
| **1286937** | chr14 | 89411246 | + | 0.362444 | 90141 | C14orf143 | 3 | 0.679 | II |
| **2242659** | chr6 | 31709491 | - | 0.359696 | 7916 | BAT2 | 8 | 0.449 | II |
| **2939635** | chr8 | 56527146 | + | 0.340987 | 114786 | XKR4 | 7 | 0.576 | II |
| **6104432** | chr20 | 44085421 | + | 0.330386 | 1E+08 | LOC100128028 | 8 | 0.492 | II |
| **1129593** | chr12 | 1.04E+08 | + | 0.323032 | 121053 | C12orf45 | 13 | 0.298 | II |
| **1061808** | chr6 | 32244524 | - | 0.318305 | 10554 | AGPAT1 | 5 | 0.623 | II |
| **1061808** | chr6 | 32244524 | - | 0.318305 | 80864 | EGFL8 | 5 | 0.623 | II |
| **644057** | chr11 | 1.2E+08 | + | 0.317557 | 2900 | GRIK4 | 6 | 0.546 | II |
| **2073818** | chr9 | 1.36E+08 | - | 0.317367 | 1E+08 | LOC100130548 | 13 | 0.428 | II |
| **3756400** | chr5 | 68701491 | - | 0.309848 | 5884 | RAD17 | 4 | 0.594 | II |
| **3756400** | chr5 | 68701491 | - | 0.309848 | 6880 | TAF9 | 4 | 0.594 | II |
| **3094609** | chr6 | 31273544 | - | 0.30767 | 253018 | HCG27 | 2 | 0.566 | II |
| **1044043** | chr6 | 32901958 | - | 0.303616 | 6891 | TAP2 | 3 | 0.727 | II |
| **7966050** | chr12 | 1.22E+08 | + | 0.301122 | 51329 | ARL6IP4 | 6 | 0.546 | II |
| **819976** | chr1 | 1411393 | + | 0.294063 | 83858 | ATAD3B | 12 | 0.46 | II |
| **8022** | chr4 | 55301183 | + | 0.286554 | 3815 | KIT | 17 | 0.318 | II |
| **13330491** | chr16 | 30012700 | + | 0.283572 | 6911 | TBX6 | 3 | 0.727 | II |
| **13330491** | chr16 | 30012700 | + | 0.283572 | 83719 | YPEL3 | 3 | 0.727 | II |
| **2228075** | chr7 | 1.28E+08 | - | 0.283349 | 3614 | IMPDH1 | 17 | 0.318 | II |
| **9299214** | chr9 | 1.15E+08 | + | 0.276942 | 246184 | CDC26 | 9 | 0.506 | II |
| **2280231** | chr11 | 47557013 | + | 0.275254 | 4722 | NDUFS3 | 11 | 0.458 | II |
| **1723484** | chr2 | 47253478 | - | 0.272429 | 805 | CALM2 | 7 | 0.505 | II |
| **2286612** | chr11 | 63750805 | - | 0.266832 | 83707 | TRPT1 | 9 | 0.506 | II |
| **2286612** | chr11 | 63750805 | - | 0.266832 | 84304 | NUDT22 | 9 | 0.506 | II |
| **9066** | chr2 | 1.05E+08 | - | 0.261664 | 64965 | MRPS9 | 9 | 0.513 | II |
| **3779794** | chr8 | 87740511 | - | 0.257398 | 54714 | CNGB3 | 18 | 0.279 | II |
| **268791** | chr5 | 72241194 | + | 0.256267 | 3842 | TNPO1 | 9 | 0.513 | II |
| **11169571** | chr12 | 49500031 | + | 0.254127 | 466 | ATF1 | 7 | 0.587 | II |
| **13181** | chr19 | 50546758 | + | 0.247735 | 2068 | ERCC2 | 10 | 0.531 | II |
| **13181** | chr19 | 50546758 | + | 0.247735 | 147700 | KLC3 | 10 | 0.531 | II |
| **11556099** | chr22 | 38247806 | + | 0.247698 | 468 | ATF4 | 9 | 0.622 | II |
| **28670911** | chr5 | 1.77E+08 | + | 0.24707 | 27166 | PRELID1 | 2 | 0.567 | II |
| **3130062** | chr6 | 31633890 | + | 0.235027 | 4795 | NFKBIL1 | 2 | 0.567 | II |
| **7953706** | chr12 | 31118101 | + | 0.232592 | 1663 | DDX11 | 7 | 0.646 | II |
| **7953706** | chr12 | 31118101 | + | 0.232592 | 440081 | DDX12 | 7 | 0.646 | II |
| **12507409** | chr4 | 1.58E+08 | + | 0.224099 | 2743 | GLRB | 8 | 0.559 | II |
| **7811** | chr18 | 3246111 | - | 0.219773 | 10627 | MYL12A | 12 | 0.56 | II |
| **2899849** | chr14 | 35859525 | + | 0.217547 | 51562 | MBIP | 9 | 0.571 | II |
| **10820739** | chr9 | 98303641 | + | 0.211798 | 8555 | CDC14B | 5 | 0.532 | II |
| **1049526** | chr6 | 33056781 | + | 0.209996 | 6046 | BRD2 | 1 | NA | II |
| **6942** | chr2 | 1.9E+08 | + | 0.205553 | 94101 | ORMDL1 | 5 | 0.532 | II |
| **6650** | chr8 | 1.21E+08 | + | 0.205162 | 28998 | MRPL13 | 4 | 0.744 | II |
| **9904590** | chr17 | 38203397 | + | 0.204831 | 28958 | CCDC56 | 9 | 0.571 | II |
| **9904590** | chr17 | 38203397 | + | 0.204831 | 124817 | CNTD1 | 9 | 0.571 | II |
| **8128** | chr1 | 1.15E+08 | - | 0.202547 | 10286 | BCAS2 | 6 | 0.718 | II |
