# Supplementary material for: dsPIG: a tool to predict imprinted genes from the deep sequencing of whole transcriptomes
Source: BMC Bioinformatics. 2012 Oct 19;13:271. doi: 10.1186/1471-2105-13-271 (PMC3497615; doi:10.1186/1471-2105-13-271)
Supplement: Additional file 4 — Table S2.The FDR values with respect to different sample sizes and allele frequencies. “ NA” means FDR could not be estimated based on our 20,000-time simulations [file 1471-2105-13-271-S4.doc]

**Table S2 The FDR values with respect to different sample sizes and allele frequencies.** “NA” means FDR could not be estimated based on our 20,000-time simulations.

| **Sample Size ^mAF^** | **0.1** | **0.2** | **0.3** | **0.4** | **0.5** |
| --- | --- | --- | --- | --- | --- |
| **1** | NA | NA | NA | NA | NA |
| **2** | 0.719 | 0.746 | NA | NA | NA |
| **3** | 0.708 | 0.752 | 0.881 | NA | NA |
| **4** | 0.742 | 0.729 | 0.747 | 0.834 | NA |
| **5** | 0.675 | 0.709 | 0.743 | 0.748 | 0.803 |
| **6** | 0.561 | 0.662 | 0.724 | 0.717 | 0.714 |
| **7** | 0.589 | 0.622 | 0.658 | 0.645 | 0.594 |
| **8** | 0.598 | 0.575 | 0.626 | 0.565 | 0.506 |
| **9** | 0.556 | 0.595 | 0.584 | 0.545 | 0.548 |
| **10** | 0.553 | 0.604 | 0.535 | 0.486 | 0.455 |
| **11** | 0.672 | 0.59 | 0.485 | 0.405 | 0.401 |
| **12** | 0.632 | 0.557 | 0.442 | 0.357 | 0.349 |
| **13** | 0.62 | 0.534 | 0.401 | 0.301 | 0.293 |
| **14** | 0.604 | 0.49 | 0.367 | 0.254 | 0.231 |
| **15** | 0.579 | 0.434 | 0.303 | 0.219 | 0.18 |
| **16** | 0.568 | 0.41 | 0.245 | 0.153 | 0.145 |
| **17** | 0.553 | 0.398 | 0.213 | 0.115 | 0.127 |
| **18** | 0.53 | 0.379 | 0.18 | 0.078 | 0.103 |
| **19** | 0.567 | 0.342 | 0.153 | 0.065 | 0.07 |
| **20** | 0.56 | 0.327 | 0.115 | 0.074 | 0.061 |
| **21** | 0.535 | 0.283 | 0.107 | 0.048 | 0.048 |
| **22** | 0.51 | 0.248 | 0.074 | 0.043 | 0.048 |
| **23** | 0.522 | 0.216 | 0.048 | 0.034 | 0.038 |
| **24** | 0.519 | 0.194 | 0.052 | 0.015 | 0.024 |
| **25** | 0.494 | 0.153 | 0.048 | 0.015 | 0.015 |
| **26** | 0.477 | 0.142 | 0.048 | 0.005 | 0.01 |
| **27** | 0.463 | 0.13 | 0.034 | 0.01 | 0.005 |
| **28** | 0.437 | 0.107 | 0.024 | 0.015 | 0.005 |
| **29** | 0.422 | 0.091 | 0.024 | 0.015 | 0 |
| **30** | 0.408 | 0.07 | 0.015 | 0.01 | 0 |
| **31** | 0.396 | 0.061 | 0.005 | 0.005 | 0 |
| **32** | 0.38 | 0.043 | 0.005 | 0.005 | 0 |
| **33** | 0.363 | 0.048 | 0.005 | 0 | 0 |
| **34** | 0.363 | 0.043 | 0.005 | 0.005 | 0 |
| **35** | 0.355 | 0.043 | 0.005 | 0 | 0 |
| **36** | 0.331 | 0.029 | 0.005 | 0 | 0 |
| **37** | 0.308 | 0.024 | 0.01 | 0 | 0 |
| **38** | 0.296 | 0.02 | 0.005 | 0 | 0 |
| **39** | 0.283 | 0.01 | 0.005 | 0 | 0 |
| **40** | 0.275 | 0.01 | 0 | 0 | 0 |
| **41** | 0.251 | 0.005 | 0 | 0 | 0 |
| **42** | 0.248 | 0 | 0 | 0 | 0 |
| **43** | 0.237 | 0.005 | 0 | 0 | 0 |
| **44** | 0.231 | 0.01 | 0 | 0 | 0 |
| **45** | 0.216 | 0.015 | 0 | 0 | 0 |
| **46** | 0.203 | 0.01 | 0 | 0 | 0 |
| **47** | 0.19 | 0.01 | 0 | 0 | 0 |
| **48** | 0.19 | 0.01 | 0 | 0 | 0 |
| **49** | 0.187 | 0.005 | 0 | 0 | 0 |
| **50** | 0.174 | 0.005 | 0 | 0 | 0 |
