# Supplementary material for: dsPIG: a tool to predict imprinted genes from the deep sequencing of whole transcriptomes
Source: BMC Bioinformatics. 2012 Oct 19;13:271. doi: 10.1186/1471-2105-13-271 (PMC3497615; doi:10.1186/1471-2105-13-271)
Supplement: Additional file 9 — R package (dsPIG, version 3.0) for Windows). [file 1471-2105-13-271-S9.zip › dsPIG/html/00Index.html]

R: deep sequencing-based Prediction of Imprinted Genes

# deep sequencing-based Prediction of Imprinted Genes

---

## Documentation for package ‘dsPIG’ version 3.0

- DESCRIPTION file.

## Help Pages

|  |  |
| --- | --- |
| AllelicCount | Count the Number of Alleles for each SNP |
| formatSNPdatabase | formatting the SNP database |
| geneinfo | Annotation data for human genes |
| genePos | Genome position data for human genes |
| ImprintedGene | Existing human imprinted genes |
| ImprintingPrediction | Identify imprinted genes on the basis of posteriors calculated for SNPs |
| mark\_genome | Generate a necessary file used by the "AllelicCount" function in the package of dsPIG |
| ProbabilityOfImprinting | Calculate the posterior probability of imprinting for each gene |
| Sam2Eland | Convert SAM format to Solexa Eland format |
| SNP4Validation | Identify genes worth further validations |
