# Supplementary material for: dsPIG: a tool to predict imprinted genes from the deep sequencing of whole transcriptomes
Source: BMC Bioinformatics. 2012 Oct 19;13:271. doi: 10.1186/1471-2105-13-271 (PMC3497615; doi:10.1186/1471-2105-13-271)
Supplement: Additional file 10 — The instruction and the sample files for the R package of dsPIG. [file 1471-2105-13-271-S10.zip › Instructions for dsPIG.pdf]

dsPIG: a Tool to Predict Imprinted Genes from the Deep Sequencing of Whole Transcriptomes

Hua Li

August 10, 2012

1 Introduction

This vignette is intended to give a rapid introduction to the commands used in the R package of dsPIG. dsPIG (deep sequencing-based Prediction of Imprinted Genes) is a Bayesian model developed to predict imprinted genes on the basis of allelic expression observed in the mRNA-Seq data of various independent human tissues. It uses known SNPs to distinguish and count the two alleles of each gene in the transcriptome of each tissue sample. With the allelic counts, it computes the posterior probability of imprinting for each gene based on each SNP. dsPIG is capable of identifying imprinted genes with high sensitivity and specificity and a low false discovery rate when the number of sequenced tissue samples is fairly large. With the prevalence of mRNA-Seq technology, dsPIG has become a useful tool for analyzing allelic expression and predicting imprinted genes. For more details on the methods being used, please refer to “dsPIG: a useful tool to predict imprinted genes from the deep sequencing of transcriptomes”, or consult Dr. Shoudan Liang.

2 Preparation

We first download the example data from http://www.shoudanliang.com/dsPIG/ and put all the files into the working directory of R. Then, we load the dsPIG package in R:

>library(dsPIG)

In this example, we have mapped mRNA-Seq data (in Solexa Eland format) from 10 independent human tissue samples. As required by the **AllelicCount** function, we first need to name these data files with “S1.txt”, “S2.txt” … “S10.txt”. To rapidly implement the functions in this package as an example, we only included 1000 mapped sequencing tags in each data file, which covered 100 SNPs. For example:

>S1=read.table(“S1.txt”, as.is=T)

>dim(S1)

[1] 1000   3

>S1[1:5,]

1	ACAGCACCCCCCAGGCAGGGACAAAGGCTCAC	chr19:558295	R
2	GAAGTCATGCACGCTATCTGATAAAGAAGAAC	chr17:451978	F
3	GAGCACTGCCCCATGCCCACCCTGTGTACCCA	chr16:573330	F
4	GAGCACACTCCCACACGCGCATGTATACGTGC	chr1:1222162	F
5	TGGGGCCGAGACTCAGGGCTCACATTGCCCCC	chr19:570889	F

Here, the first column is the nucleotides of the sequence read, chr3:187821633 is the chromosome and position the read has been mapped to. "F" indicates forward strand and "R" indicates reverse strand. All 10 data files have the same Solexa Eland format as shown above.

If the data files are in SAM format, we can convert them to Solexa Eland format by using the **Sam2Eland** function. For example, to convert “SamFormatExample.txt”, we use:

>Sam2Eland("SamFormatExample.txt", "ElandOut.txt")

This function generates a file called “ElandOut.txt” in the working directory, which is in Solexa Eland format.

SNP information needs to be retrieved before computing the posterior probability of imprinting. We have two SNP files for the 100 SNPs covered by the sequence reads:

>SNPdatabase=read.delim("SNPdatabaseEG.txt", as.is=T)

>dim(SNPdatabase)

[1] 100 9

>SNPdatabase[1:5,]

	chrom	chromStart	chromEnd	name	strand	observed	avHet	avHetSE	class
1	chr17	193187	193188	rs4247114	-	C/T	0.397883	0.201571	single
2	chr16	1347441	1347442	rs6600137	+	A/G	0.095791	0.196773	single
3	chr9	835563	835564	rs16924776	+	C/T	0.403200	0.197559	single
4	chr10	1139389	1139390	rs7915650	+	A/C	0.240250	0.249810	single
5	chr7	983473	983474	rs10272099	+	C/T	0.494952	0.049985	single

Here, "chrom", "chromStart" and "chromEnd" are the coordinate of the SNP; "name" is the name of the SNP in the form of "rs#"; "strand" is the strand the SNP is on; "observed" is the observed alleles of the SNP; "avHet" and "avHetSE" are the average heterogezcosity and the standard error of avHet. The SNP builds under HG18 or HG19 all have these fields with exactly the same names.

>AlleleFreqFile=read.delim("Allele_Freq.txt", as.is=T)

>dim(AlleleFreqFile)

[1] 100 8

>AlleleFreqFile[1:5,]

	SNP	chr	position	str	allele_1	frequency_1	allele_2	frequency_2
1	rs4247114	chr17	193187	-	C	0.049	T	0.951
2	rs6600137	chr16	1347441	+	A	0.080	G	0.920
3	rs16924776	chr9	835563	+	T	0.874	C	0.126
4	rs7915650	chr10	1139389	+	C	0.792	A	0.208
5	rs10272099	chr7	983473	+	T	0.677	C	0.323

The first column is the SNP ID; the second, third and fourth columns are the chromosome, position and strand the SNP is on; the fifth and seventh columns are the observed two alleles; the sixth and eighth column are the corresponding allele frequencies.

We also prepared a file that maps SNP IDs (rs#) to Entrez Gene IDs and gene symbols:

>SNP2Gene=read.delim("SNP2Gene.txt", as.is=T)

>dim(SNP2Gene)

[1] 100 3

>SNP2Gene[1:5,]

	SNP_ID	Entrez_ID	Symbol
1	4247114	9501	RPH3AL
2	6600137	84572	GNPTG
3	16924776	1761	DMRT1
4	7915650	22884	WDR37
5	10272099	90639	COX19

3 Results

To use dsPIG to compute the posterior probability of imprinting, we first use the **mark_genome** to create a file called "mark_genome.txt" in the working directory, which is required by **AllelicCount**. The data files (i.e., "S1.txt", "S2.txt" … "S10.txt") used by **mark_genome** must be in the current working directory.

> mark_genome(nsample=10)

Here, nsample is the total number of files from which the mapped mRNA-Seq data (in Solexa Eland format) are to be read from. In this example, nsample=10.

Now we use the **formatSNPdatabase** function to convert the SNP database into a fixed format that can be used by the **AllelicCount** function.

```
>formatSNPdatabase("SNPdatabaseEG.txt", "SNPdatabaseEGformat.txt")
```

This function generates a file called “SNPdatabaseEGformat.txt” in the current working directory, which is then used by **AllelicCount**. The **AllelicCount** function reads the mapped mRNA-Seq data in Solexa Eland format, and for each input file, it generates a corresponding output file with a prefix "out_" that counts the number of times each allele appears in the transcriptome.

```
>AllelicCount(nsample=10, SNPdatabase="SNPdatabaseEGformat.txt", samplefile= c("S1.txt", "S2.txt", "S3.txt", "S4.txt", "S5.txt", "S6.txt", "S7.txt", "S8.txt", "S9.txt", "S10.txt"), nmax=100000)
```

```
>Out1=read.delim(“out_S1.txt”, as.is=T)
```

```
>dim(Out1)
```

```
[1] 100   5
```

```
>Out1[1:5,]
```

```
  A  C G  T      SNP
1 0 10 0  0  rs4247114
2 3  0 7  0  rs6600137
3 0  0 0 10  rs16924776
4 4  6 0  0  rs7915650
5 0  0 0 10  rs10272099
```

The **ProbabilityOfImprinting** function uses the output files from **AllelicCount** and the allele frequency data to calculate the posterior probability of imprinting for each gene based on each SNP. In this example, it uses “out_S1.txt”, “out_S2.txt” … “out_S10.txt” and “Allele_Freq.txt” to calculate the posterior probability.

```
>Result=ProbabilityOfImprinting(AlleleFreqFile="Allele_Freq.txt", Samples= c("out_S1.txt", "out_S2.txt", "out_S3.txt", "out_S4.txt", "out_S5.txt", "out_S6.txt", "out_S7.txt", "out_S8.txt", "out_S9.txt", "out_S10.txt"), SNP2Gene='SNP2Gene.txt', error=0.02, Imp=0.01, QS=0.9)
```

```
>dim(Result)
```

[1] 100   12

```
> Result[1:5,]
```

	RS	chr	position	str	allele_1	frequency_1	allele_2	frequency_2	Posterior	geneID	Symbol	SS
1	4247114	chr17	193187	-	C	0.049	T	0.951	1.0000e+00	9501	RPH3AL	10
2	6600137	chr16	1347441	+	A	0.080	G	0.920	8.5128e-36	84572	GNPTG	10
3	16924776	chr9	835563	+	T	0.874	C	0.126	3.7284e-02	1761	DMRT1	10
4	7915650	chr10	1139389	+	C	0.792	A	0.208	9.3084e-35	22884	WDR37	10
5	10272099	chr7	983473	+	T	0.677	C	0.323	3.3102e-01	90639	COX19	10

The first column is the SNP ID (without the prefix “rs#”) with “RS” as the column name. The 10th column is the Entrez Gene ID, with “geneID” as the column name. The rest of the column names are the same as above.

We then use the **ImprintingPrediction** function to predict imprinting genes (the input file usually comes from the **ProbabilityOfImprinting** function).

```
>Prediction=ImprintingPrediction(GeneWithPosterior=Result, threshold=0.2)
```

```
>dim(Prediction)
```

[1] 18   11

```
>Prediction[1:5,]
```

	RS	chr	position	str	allele_1	frequency_1	allele_2	frequency_2	Posterior	geneID	SS
1	4247114	chr17	193187	-	C	0.049	T	0.951	1.0000000	9501	10
5	10272099	chr7	983473	+	T	0.677	C	0.323	0.3310159	90639	10
16	1128321	chr11	234170	+	T	0.230	C	0.770	0.9999562	5719	10

23	1132356	chr16	1334507	+	A	0.098	C	0.902	1.0000000	8938	10
25	3752242	chr19	1004676	+	G	0.578	A	0.422	0.7052541	10347	10

Finally, we use the **SNP4Validation** function to identify genes that are worth further (experimental) validations. The input file for **SNP4Validation** is usually the output file from the **ImprintingPrediction** function.

>Validation=SNP4Validation(Prediction, mAF=0.184, distance=2000000)

>dim(Validation)

[1] 14 11

>Validation[1:5,]

	RS	chr	position	str	allele_1	frequency_1	allele_2	frequency_2	Posterior	geneID	SS
5	10272099	chr7	983473	+	T	0.677	C	0.323	0.3310159	90639	10
16	1128321	chr11	234170	+	T	0.230	C	0.770	0.9999562	5719	10
25	3752242	chr19	1004676	+	G	0.578	A	0.422	0.7052541	10347	10
26	2562147	chr16	54534	+	A	0.243	G	0.757	0.9999245	64285	10
29	710925	chr16	573354	+	G	0.535	A	0.465	0.8379161	9091	10

4 Conclusions

The R package of dsPIG has provided tools to analyze allelic expression and predict imprinted genes. Currently, this package emphasizes processing of mapped mRNA-Seq reads in Solexa Eland format. Including support for more file types from additional manufactures is the development priority in the near future.
