# Supplementary material for: dsPIG: a tool to predict imprinted genes from the deep sequencing of whole transcriptomes
Source: BMC Bioinformatics. 2012 Oct 19;13:271. doi: 10.1186/1471-2105-13-271 (PMC3497615; doi:10.1186/1471-2105-13-271)
Supplement: Additional file 6 — Figure S4. Functional enrichment analysis of the 57 genes and the 94 genes in Ingenuity® Pathway Analysis. (a) Comparison of enrichment in Bio Functions between the two gene lists. (b) Comparison of enrichment in Canonical Pathways between the two gene lists. The height of each bar in (a) and (b) represents the logartihm (10-based) transformed p-values calculated from Fisher’s exact test. In (a), the horizontal yellow line is the threshold [i.e., -log10(0.05)] above which bars (p-values) were considered significant; in (b), there is no bar above the threshold which is not shown here. (Figure S4 is located in a separate PDF file: “Fig. S4.pdf”.) [file 1471-2105-13-271-S6.pdf]

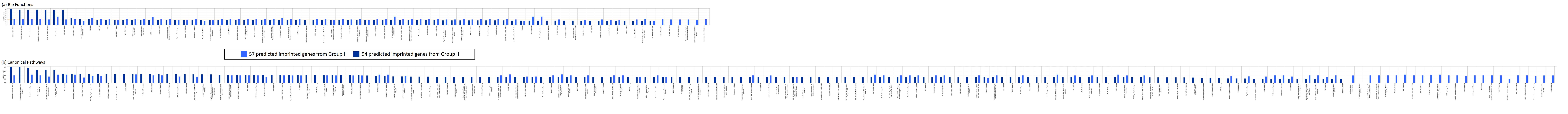

(a) Bio Functions
57 predicted imprinted genes from Group I
94 predicted imprinted genes from Group II
(b) Canonical Pathways
